# Splicing Endonuclease Is an Important Player in rRNA and tRNA Maturation in Archaea

**DOI:** 10.3389/fmicb.2020.594838

**Published:** 2020-11-20

**Authors:** Thandi S. Schwarz, Sarah J. Berkemer, Stephan H. Bernhart, Matthias Weiß, Sébastien Ferreira-Cerca, Peter F. Stadler, Anita Marchfelder

**Affiliations:** ^1^Biologie II, Ulm University, Ulm, Germany; ^2^Bioinformatics, Department of Computer Science, Leipzig University, Leipzig, Germany; ^3^Max Planck Institute for Mathematics in the Sciences, Leipzig, Germany; ^4^Competence Center for Scalable Data Services and Solutions, Leipzig University, Leipzig, Germany; ^5^Interdisciplinary Center for Bioinformatics, Leipzig University, Leipzig, Germany; ^6^Regensburg Center for Biochemistry, Biochemistry III – Institute of Biochemistry, Genetics and Microbiology, University of Regensburg, Regensburg, Germany; ^7^German Centre for Integrative Biodiversity Research (iDiv) Halle-Jena-Leipzig, Leipzig, Germany; ^8^Research Center for Civilization Diseases, Leipzig University, Leipzig, Germany; ^9^Facultad de Ciencias, Universidad Nacional de Colombia, Bogotá, Colombia; ^10^Institute for Theoretical Chemistry, University of Vienna, Vienna, Austria; ^11^Center for RNA in Technology and Health, University of Copenhagen, Frederiksberg, Denmark; ^12^Santa Fe Institute, Santa Fe, NM, United States

**Keywords:** splicing endonuclease, tRNA intron, rRNA intron, tRNA processing, rRNA processing

## Abstract

In all three domains of life, tRNA genes contain introns that must be removed to yield functional tRNA. In archaea and eukarya, the first step of this process is catalyzed by a splicing endonuclease. The consensus structure recognized by the splicing endonuclease is a bulge-helix-bulge (BHB) motif which is also found in rRNA precursors. So far, a systematic analysis to identify all biological substrates of the splicing endonuclease has not been carried out. In this study, we employed CRISPRi to repress expression of the splicing endonuclease in the archaeon *Haloferax volcanii* to identify all substrates of this enzyme. Expression of the splicing endonuclease was reduced to 1% of its normal level, resulting in a significant extension of lag phase in *H. volcanii* growth. In the repression strain, 41 genes were down-regulated and 102 were up-regulated. As an additional approach in identifying new substrates of the splicing endonuclease, we isolated and sequenced circular RNAs, which identified excised introns removed from tRNA and rRNA precursors as well as from the 5′ UTR of the gene HVO_1309. *In vitro* processing assays showed that the BHB sites in the 5′ UTR of HVO_1309 and in a 16S rRNA-like precursor are processed by the recombinant splicing endonuclease. The splicing endonuclease is therefore an important player in RNA maturation in archaea.

## Introduction

**FIGURE 1:**
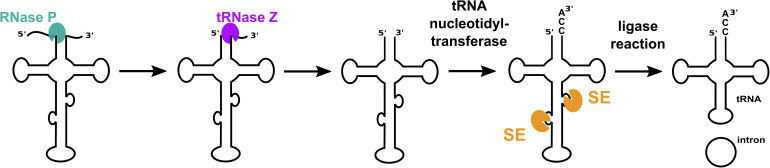
tRNA maturation in archaea. Functional mature tRNAs are obtained after several processing steps. The 5′ leader and 3′ trailer sequences are removed by the endonucleases RNase P and tRNase Z. A terminal CCA triplet is added to the 3′ end by tRNA nucleotidyl transferase. Introns are removed by a splicing endonuclease (SE), exons are ligated, and the intron is circularized by an RNA ligase. The order of the processing steps can differ from organism to organism.

tRNA molecules are key players within cells since they translate genetic information into protein. Generation of functional tRNA molecules requires a plethora of processing steps starting with the removal of 5′ leader and 3′ trailer sequences from pre-tRNA [for a review see (Clouet-d’Orval et al., 2018; Figure 1)]. Some tRNA genes contain introns that must also be removed from precursor RNA to yield mature functional tRNAs.

While some bacterial tRNA genes contain self-splicing group I introns, archaeal and eukaryotic tRNA introns are removed by proteins. The initial step of intron removal in eukaryotes and archaea is catalyzed by an RNA splicing endonuclease. The resulting splice products are ligated by a tRNA ligase, thereby generating mature tRNA as well as a circular intron (Figure 1). In some organisms, (e.g., *S. cerevisiae*) the enzyme 2′-phosphotransferase is required to remove the 2′ phosphate at the ligation junction to yield mature tRNA (Steiger et al., 2005). Structures for archaeal splicing endonucleases have been determined. Four different quaternary structures have emerged: an α_4_ homotetramer from *Methanocaldococcus jannaschii* (Lykke-Andersen and Garrett, 1997; Li et al., 1998), an α_2_ homodimer from *H. volcanii* (Kleman-Leyer et al., 1997), a (αβ)_2_ dimer of heterodimers from *Nanoarchaeum equitans* (Mitchell et al., 2009) and, more recently, an irregular hexamer, ε_2_ from Candidatus *Microarchaeum acidiphilum* (Fujishima et al., 2011). Each of the two ε substructures of the ε_2_ complex consists of three proteins: a catalytic α subunit, a β subunit and a pseudocatalytic α subunit, denoted as α^p^, forming an irregular homodimeric “six-unit” architecture [ε = (α^p^-α-β)].

**FIGURE 2:**
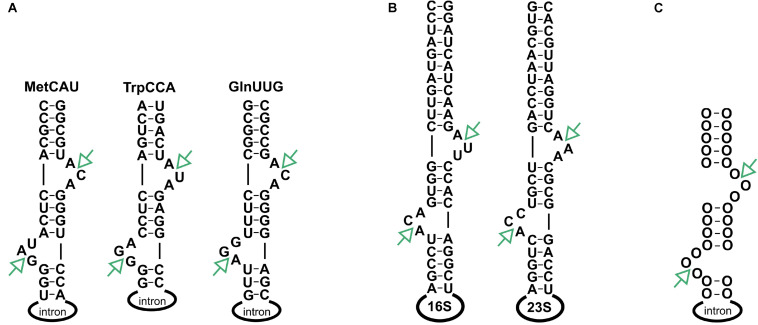
Location of BHB motifs. **(A)** Three tRNAs contain an intron with the hBHBh′ motif forming at the exon-intron boundary. **(B)** hBHBh′ motifs are also flanking the 16S and 23S rRNAs. **(C)** General structure of the hBHBh′ motif. SE processing sites are indicated by green arrows.

Despite structural differences, all archaeal splicing endonucleases recognize a conserved structure, referred to as a bulge-helix-bulge motif (BHB), which features a four-nucleotide helix flanked by two bulges, each comprised of three nucleotides (Figure 2; Marck and Grosjean, 2003; Fujishima et al., 2011). The heterodimeric (αβ)_2_ form of the splicing endonuclease which is found mainly in the TACK^1^ superphylum accepts a broader substrate spectrum with BHB motifs that contain various bulge lengths (Watanabe et al., 2002; Clouet-d’Orval et al., 2018). Notably, non-canonical BHB motifs are prevalent in the crenarchaea (Marck and Grosjean, 2003; Fujishima et al., 2011). Similarly, the splicing endonuclease ε_2_ found in ARMAN^2^ archaea has a broad substrate spectrum (Fujishima et al., 2011; Clouet-d’Orval et al., 2018). The BHB motif is usually found in the tRNA anticodon arm but can also be present in other locations in the tRNA (Marck and Grosjean, 2003). In addition, the BHB motif was found in rRNAs (Kjems and Garrett, 1991; Qi et al., 2020) and in one mRNA (Watanabe et al., 2002). In another study (Tang et al., 2002), it was suggested that circular 16S and 23S pre-rRNAs may be generated upon cleavage at the BHB site and subsequent ligation of exposed ends. Indeed, the presence of circular pre-rRNAs was confirmed in subsequent studies (Danan et al., 2012; Jüttner et al., 2020); however the enzyme catalyzing the reaction *in vivo* was unknown. More recently, using *in vitro* methods, it was shown that a splicing endonuclease catalyses this reaction in methanoarchaea (Qi et al., 2020).

*Haloferax volcanii* is a halophilic archaeon belonging to the kingdom euryarchaeal. *H. volcanii* contains a α_2_ type splicing endonuclease, which strictly requires a hBHBh′^3^ motif and will not cleave any other BHB (Thompson and Daniels, 1990). The *Haloferax* genome encodes three intron containing tRNA genes with intron lengths of 31 to 103 nucleotides: tRNA^Trp^_CCA_: 103, tRNA^Met^_CAT_: 75, tRNA^Gln^_TTG_: 31, respectively. Moreover, the precursor RNAs for 16S rRNA and 23S rRNAs both contain a BHB motif (Figure 2). Splicing endonuclease processing at these BHB motifs would release the rRNAs from the multicistronic pre-rRNA. The *Haloferax* splicing endonuclease has been investigated in detail and was shown to be active at low salt concentrations *in vitro* (Thompson and Daniels, 1990). This is surprising since *Haloferax* contains high intracellular salt concentrations (Mullakhanbhai and Larsen, 1975; Oren, 2008). Substrate specificities for the *Haloferax* endonuclease have also been reported (Thompson and Daniels, 1990; Armbruster and Daniels, 1997). A potential cellular function for the excised intron was investigated for tRNA^Trp^ in *Haloferax* (Clouet d’Orval et al., 2001; Singh et al., 2004). The intron for this tRNA contains a box C/D sRNA that is required to direct 2′-*O*- methylation of nucleotides C34 and U39 in tRNA^Trp^. Removal of the intron part of the tRNA gene has no effect on cell viability under standard conditions, suggesting that the intron is not maintained due to the sRNA (Joardar et al., 2008). To date, no other systematic analyses have been performed *in vivo* to identify all cellular substrates and functions of the archaeal splicing endonucleases. Moreover, very few ribonucleases have been functionally characterized in archaea. The archaeal tRNA 3′ end processing enzyme tRNase Z has been identified (Späth et al., 2008) and shown to catalyze not only tRNA 3′ processing but also 5′ end maturation of the 5S rRNA (Hölzle et al., 2008). To investigate whether the splicing endonuclease also processes substrates other than tRNAs, we set out to determine all biological substrates of the splicing endonuclease in *Haloferax*. Here, we use a systematic analysis of splicing endonuclease substrates *in vivo* to show that, in addition to the known tRNA substrates, one additional mRNA and the predicted rRNA substrates are cleaved by the splicing endonuclease. These *in vivo* observations were confirmed by *in vitro* assays.

## Results

### RNA Splicing Endonuclease From *Haloferax volcanii*

**FIGURE 3:**
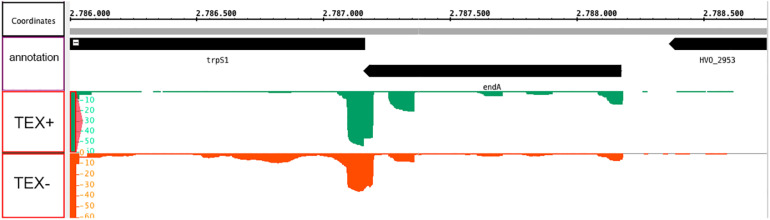
Genomic location of the *endA* gene and transcription start site (TSS) analysis. The *endA* gene is encoded on the minus strand and overlaps the downstream *trpS1* gene by four nucleotides. dRNA-Seq analysis was performed to identify TSSs in *H. volcanii* (Babski et al., 2016). Green signals: the isolated RNA was treated with terminal exonuclease (+TEX), red signals: the RNA was not treated with TEX (–TEX). Comparison of reads from both fractions allowed us to determine the TSSs. The genome sequence is shown in the middle (with the genomic location in nucleotides, annotated genes are shown in black bars). Green (+TEX) and red (–TEX) regions represent dRNA-Seq reads, and the height corresponds to the coverage (shown at the left).

*Haloferax volcanii* encodes an RNA splicing endonuclease (HVO_2952, *endA*) in a bicistronic operon together with a tryptophanyl-tRNA synthetase (HVO_2951, *trpS1*) (Figure 3), the two genes overlap by four nucleotides. Both the prokaryotic operon database (Taboada et al., 2012) and our transcriptome (Berkemer et al., 2020), TSS (Babski et al., 2016) and transcription termination site (TTS) data (Berkemer et al., 2020) confirm this genomic organization. According to our TSS studies (Babski et al., 2016), the gene for the splicing endonuclease seems to contain two promoters for the downstream *trpS1* gene (Figure 3), therefore the *trpS1* gene could be transcribed independently from the *endA* gene. TSS data also show, that transcription from the *endA* promoter is comparatively low. In addition, the *endA* mRNA is not detectable on northern blots (data not shown). But proteome data clearly show that the splicing endonuclease is present in the proteome (Jevtić et al., 2019; Schulze et al., 2020).

To investigate the biological function of the splicing endonuclease, we aimed to repress its expression using CRISPR interference (CRISPRi) (Stachler and Marchfelder, 2016; Stachler et al., 2019). The CRISPRi approach uses the endogenous CRISPR-Cas system to repress transcription (Stachler and Marchfelder, 2016; Stachler et al., 2019). The Cas protein complex Cascade is guided by a crRNA to the promoter region or transcription start site leading to hindering transcription initiation. If we repress transcription initiation at the *endA* promoter, the promoters for *trpS1* located in the *endA* gene are still present and able to initiate transcription of the *trpS1* gene.

### Knockdown of the *endA* Gene Results in Growth Defects and Defects in tRNA Splicing

**FIGURE 4:**
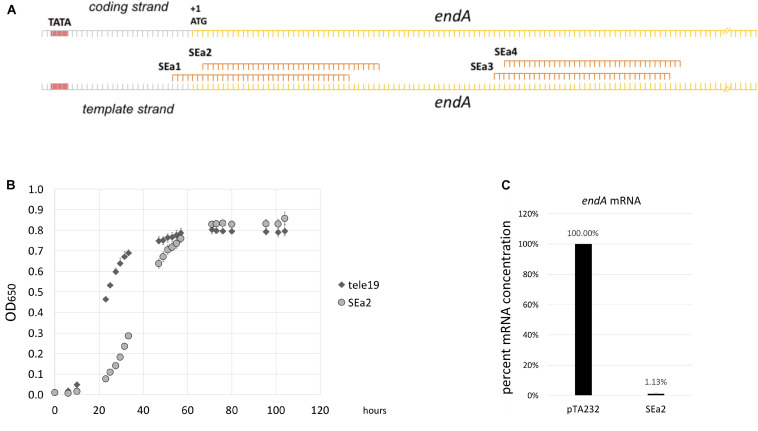
CRISPRi against the *endA* gene results in reduced *endA* mRNA concentrations. **(A)** Location of crRNAs. Four different crRNAs (SEa1-SEa4) were designed against the *endA* transcription start site and beginning of the ORF. The promoter is indicated with a red box and TATA; the transcription start site is indicated with +1; the *endA* ORF is indicated in yellow and the ATG is also shown. TSS and the A of the ATG are identical, showing that the *endA* mRNA is leaderless. For a more detailed view see Supplementary Figure 1. **(B)** Expression of crRNA SEa2 influences growth. Expression of crRNA SEa2 results in a severe lag phase (light gray circles, SEa2). As a control, a crRNA that does not bind any target in the cell was expressed (dark gray diamonds, tele19). **(C)** Reduction of *endA* mRNA concentrations upon expression of crRNA SEa2. qRT-PCR was performed to measure the concentrations of *endA* mRNA in wild type (column pTA232, cells HV30 × pTA232) and CRISPRi cells (column SEa2, cells HV30 × pTA232SEa2). The mRNA concentration in wild type cells was set to 100%. Repression with CRISPRi reduces the mRNA concentration to 1.1% (±0.5) of that of the wild type.

Transcription start sites of *Haloferax* were determined in a previous work, allowing the identification of upstream located promoter regions (Babski et al., 2016). We designed four crRNAs to target the promoter, the transcription start site and open reading frame of the *endA* gene (Figure 4A). The *Haloferax* strain HV30 was transformed with plasmids expressing these crRNAs (Stachler and Marchfelder, 2016; Stachler et al., 2019), and the resulting transformants were analyzed with respect to their growth behavior, showing that the expression of crRNA SEa2 had the strongest effect on growth (Figure 4B and Supplementary Figure 2). Cells expressing this crRNA showed a severe growth defect. RNA was extracted from these cells to determine how strong the *endA* mRNA was repressed. Since the amount of *endA* mRNA present in the cell was too low to use northern blots we used quantitative RT-PCR (qRT-PCR) to compare mRNA concentrations (Figure 4C).

**FIGURE 5:**
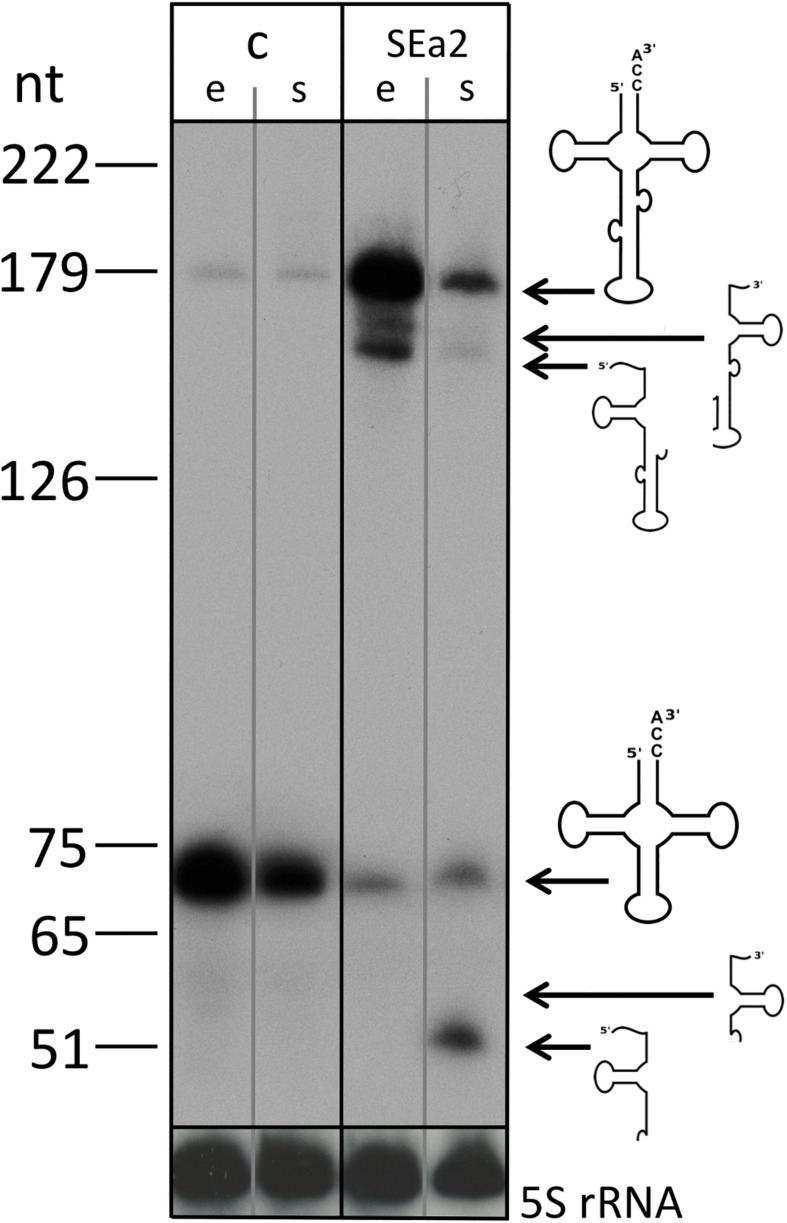
tRNA maturation is severely impeded in CRISPRi cells. RNA was isolated from control cells (lane c, HV30 × pTA232) and cells expressing crRNA SEa2 (lane SEa2, HV30 × pTA232SEa2) (lanes e: cells were grown to exponential phase, lanes s: cells were grown to stationary phase). RNA was separated by 8% PAGE and transferred to nylon membranes. Blots were hybridized with a probe against the tRNA intron that also detects the mature tRNA (upper panel; lower panel: the same blot was hybridized with a probe against the 5S rRNA). Expression of crRNA SEa2 results in accumulation of unspliced tRNA and processing intermediates. Only very small amounts of mature tRNA can be detected. A DNA size marker is given at the left. Precursors, processing intermediates and the mature product are shown schematically on the right.

Expression of crRNA SEa2 reduces the *endA* mRNA concentration down to 1.1% (±0.5%) of its wild type level in the exponential phase. To determine the effect of the lower expression of *endA* on the maturation of the intron containing tRNAs, northern blots were performed to detect the tRNA^Trp^ precursors, processing intermediates and mature form. Northern blot analyses clearly showed that the expression of crRNA SEa2 results in an accumulation of unspliced tRNAs (Figure 5).

### Repression of the Splicing Endonuclease Changes the Relative Abundances of (Pre-) Ribosomal RNA

**FIGURE 6:**
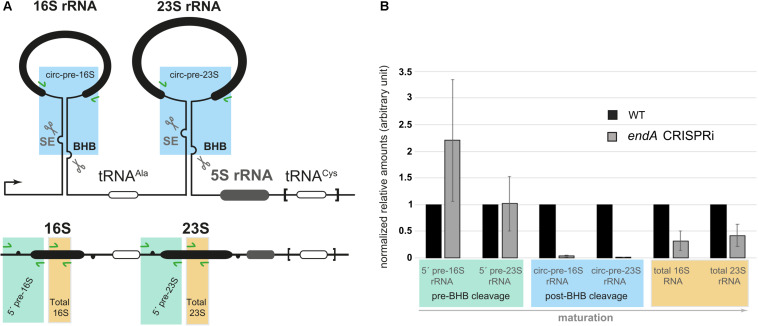
Analysis of pre-rRNA maturation in cells depleted of the RNA splicing endonuclease. **(A)** Schematic representation of the *H. volcanii* rDNA operon. One of the two rDNA operons (operon A: HVO_3038-HVO_3042 and flanking regions) present in *H. volcanii* and characterized by an additional tRNA^Cys^ gene at its 3′ end is schematically depicted. The inverted repeats flanking the mature 16S/23S rRNAs establish double stranded RNA regions containing bulge-helix-bulge (BHB) motifs, putative substrates for the splicing endonuclease (SE, scissors). Primers used for quantitative RT-PCR analysis of total 16/23S rRNAs (shown in orange), pre-circular 16S/23S rRNAs (post-BHB cleavage pre-rRNA species, blue) and 5′ pre-16S/23S rRNAs containing regions upstream of the respective 5′ bulge (pre-BHB cleavage pre-rRNA species, green) are indicated (green arrows). **(B)** Pre-rRNA maturation profile of cells depleted of SE analyzed by qRT-PCR.

Ribosomal precursor RNAs in *H. volcanii* contain two BHB motifs (Figure 6A). Recently, the structural integrity of these BHB motifs has been shown to be required for the synthesis of archaeal specific circular-pre-rRNA intermediates and mature rRNA (Jüttner et al., 2020). The presence and structure of this motif within the ribosomal processing stems is conserved in most archaea analyzed so far (Jüttner et al., 2020; Qi et al., 2020). The effect on the overall relative abundances of (pre-)rRNA in *endA* CRISPRi cells was investigated by qRT-PCR combining different sets of primers, which showed that pre-rRNAs that are not cleaved at the BHB motif slightly accumulate (Figure 6B). In addition, the amount of the circular pre-rRNAs normally cleaved at the BHB motif and circularized by the ligase is strongly decreased, showing that cleavage of the BHB motif by the splicing endonuclease (SE) is impaired. Furthermore, the consequence of *endA* repression is an overall reduced amount of total 16S and 23S rRNAs in CRISPRi cells (Figure 6). Taken together, these results established that the splicing endonuclease is essential for efficient rRNA maturation *in vivo*.

### The *trpS1* Gene Is Individually Expressed

**FIGURE 7:**
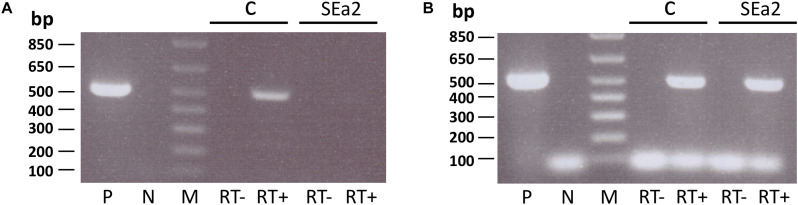
*trpS1* can be transcribed independently from *endA*. RNA was isolated from control cells (HV30 × pTA232, lanes c) and cells expressing crRNA SEa2 (HV30 × pTA232SEa2, lanes SEa2). RNAs were reverse transcribed using random hexamer primers. PCR was performed using primers spanning the overlapping region of *endA* and *trpS1* **(A)** or a part of *trpS1* **(B)**. PCR products were resolved on a 0.8% agarose gel. P: Amplification from HV30 gDNA as a PCR control. N: control without the addition of template DNA. M: 1 kb plus DNA ladder, with sizes given on the left. RT–, isolated RNA was added to exclude DNA contamination; RT+, cDNA template was added (from control cells or CRISPRi cells). Signals below 100 bp in panel B are derived from primers.

Transcriptome data show that the *trpS1* gene encoded downstream of *endA* is only slightly affected in the *endA* CRISPRi strain and is not down-regulated but slightly up-regulated (up-regulation 0.8, data not shown). Thus, down-regulation of transcription at the promoter upstream of *endA* does not repress expression of TrpS1. The promoters for *trpS1* detected in the *endA* gene (Figure 3) seem to be sufficient for adequate TrpS1 expression. To investigate whether *endA* and *trpS1* are transcribed into a bicistronic mRNA, we performed RT-PCR using primers targeting the *endA* gene and the *trpS1* gene (Figure 7A). A bicistronic mRNA was found in RNA from wild type cells; however, only low amounts of such an mRNA were present in RNA from *endA* CRISPRi cells (Figure 7B).

### Reduction of Splicing Endonuclease Expression Results in Changes in the Transcriptome

To investigate the impact of splicing endonuclease repression on the cell, we compared the transcriptomes of wild type and *endA* CRISPRi cells. RNA was isolated from triplicates of both strains, and cDNA libraries were generated and subjected to RNA-Seq (for details see Material and Methods). The resulting sequencing data were investigated, and DESeq2 (Love et al., 2014) was used to test for differentially expressed genes in *endA* CRISPRi cells in comparison to the wild type cells. Differential expression was tested for all annotated genes of *Haloferax*. Altogether, 102 genes were found to be up-regulated and 41 were down-regulated (when a threshold of log_2_: 2/−2 was applied) (Tables 1, 2 and Supplementary Tables 1, 2).

**TABLE 1:** Genes down-regulated in the *endA* CRISPRi strain.

Gene	log_2_	Annotation^a^
HVO_2952	−7,9	tRNA splicing endonuclease
HVO_0575	−4,7	Homolog to NAD-dependent epimerase/dehydratase
HVO_2519_T	−4,6	tRNA^Met^ (contains an intron)
HVO_2566	−3,8	tRNA^Gly^
HVO_0864_T	−3,5	tRNA^Gln^ (contains an intron)
HVO_B0149	−3,3	Oleate hydratase
HVO_1800	−2,9	Hypothetical protein
HVO_1561	−2,9	Conserved hypothetical protein
HVO_2804_T	−2,9	tRNA^Thr^
HVO_1276_T	−2,7	tRNA^Trp^ (contains an intron)

*The 10 most down-regulated genes are shown. The gene coding for the splicing endonuclease is the most down-regulated gene. All three tRNAs with introns are among the 10 most down-regulated genes and three genes encoding proteins of unknown function. Column “gene”: HVO gene number; column log_2_: log_2_ fold change; column “annotation”: gene annotation. ^*a*^Annotation according to HaloLex (Pfeiffer et al., 2008).*

**TABLE 2:** Genes up-regulated in the *endA* CRISPRi strain.

Gene	log_2_	Annotation^a^
HVO_1696	7.0	L-lactate permease
HVO_B0044	4.8	Siderophore biosynthesis protein, IucA
HVO_B0042	4.7	Probable 1,3-diaminopropane N-3-monooxygenase, IucD
HVO_B0045	4.5	Diaminobutyrate decarboxylase
HVO_1228	4.2	DUF5059 domain
HVO_B0043	4.1	Probable N4-hydroxy-1-aminopropane O-acetyltransferase, IucB
HVO_1697	4.1	FAD-dependent oxidoreductase (GlcD/DLD_GlcF/GlpC domain fusion protein)
HVO_B0047	4.0	ABC-type transport system periplasmic substrate-binding protein (probable substrate iron-III)
HVO_B0046	3.9	Diaminobutyrate decarboxylase diaminobutyrate-2-oxoglutarate aminotransferase
HVO_B0197	3.8	ABC-type transport system periplasmic substrate-binding protein (probable substrate iron-III)

*The 10 most up-regulated genes are shown. The gene HVO_1696, coding for an L-lactate permease is the most up-regulated gene. Six of the most up-regulated genes are located in a cluster on pHV3 (HVO_B0042-HVO_B0047) and are related to iron metabolism. Column “gene”: HVO gene number; column log_2_: log_2_ fold change; column “annotation”: gene annotation. ^*a*^Annotation according to HaloLex (Pfeiffer et al., 2008).*

The transcriptome data clearly confirm down-regulation of the splicing endonuclease, since it is the most down-regulated gene. All three intron-containing tRNAs were also down-regulated (Table 1 and Supplementary Table 1). In addition to the intron-containing tRNAs, seven other tRNAs were likewise down-regulated (Supplementary Table 1). The rRNAs were not included in the analysis since our RNA-Seq protocol included an rRNA removal step.

The 10 down-regulated tRNAs all are decoding preferentially used mRNA codons according to the codon bias determined for *Haloferax* (Nakamura and Sugiura, 2007)^4^, which might lead together with the reduced rRNA production to a general decrease in protein synthesis. The other 31 down-regulated genes included 17 genes coding for proteins and RNAs with unknown function (Supplementary Table 1).

The most up-regulated gene encodes L-lactate permease. This gene is encoded in a bicistronic operon together with the gene for FAD-dependent oxidoreductase (HVO_1697), which is also up-regulated. Six of the most up-regulated genes are located in a cluster on pHV3 (HVO_B0042-HVO_B0047) and are related to iron metabolism. Altogether, 11 transport related proteins and 22 genes coding for proteins and RNAs of unknown functions were up-regulated (Supplementary Table 2).

### Circular RNAs That Are Products of the SE Reaction

**FIGURE 8:**
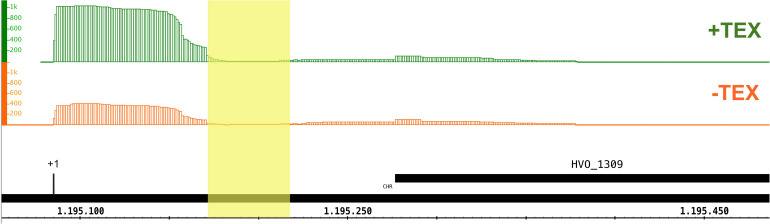
An intron is located in the 5′ UTR of HVO_1309. CircRNA-seq identified a circular RNA that is flanked by a BHB motif derived from the 5′UTR of the HVO_1309 gene. The position of the intron is shaded in yellow. The TSS is indicated by a black line and +1. TSSs were identified by dRNA-Seq analysis (Babski et al., 2016). Green: RNA treated with terminal exonuclease (+TEX), red: RNA not treated with TEX (–TEX). The height of the regions corresponds to the coverage of dRNA-Seq reads (read numbers are given at the left). Genome coordinates are indicated in nucleotides at the bottom. The HVO_1309 gene is shown as a black bar.

As an additional approach to identify all substrates of the splicing endonuclease we enriched and sequenced the circular RNAs of *H. volcanii* (circRNA-seq). The SE cleaves its substrates at the BHB motifs, and the resulting intron is circularized by a ligase. Thus, circRNA-seq identifies among others, the circular splicing products of the SE. Altogether, seven circRNAs flanked by a BHB motif were identified (Table 3). Two belong to the known tRNA and four to the predicted rRNA SE substrates; one is newly identified. The circularized intron of the third tRNA, tRNA^Gln^_TTG_, was not found, which might be due to its short length (31 nucleotides). It has been reported previously that the circRNA-seq method has a bias against short RNAs (Danan et al., 2012). Analyses of the intron position in the newly identified substrate showed that it is located in the 5′ UTR of HVO_1309 (Figure 8). HVO_1309 codes for a peptidase from the M24 family protein and transcriptome data show that HVO_1309 is down-regulated in *endA* CRISPRi cells (Supplementary Table 1). This suggests that splicing might be required for efficient expression or RNA stability.

**TABLE 3:** Circular RNAs Identified by circRNA-seq.

Left site	Right site	Intron length	Folding energy	Gene
1,195,172	1,195,218	46	−14.9	5′ UTR HVO_1309
2,384,876	2,384,951	75	−11.6	tRNA^Met^_CAT_ (intron)
1,165,777	1,165,880	103	−9.6	tRNA^Trp^ (intron)
2,770,085	2,771,742	1658	−11.8	16S rRNA
1,598,083	1,599,741	1658	−11.8	16S rRNA
1,600,017	1,602,996	2979	−14.2	23S rRNA
2,766,828	2,769,808	2980	−12.8	23S rRNA

*Seven circRNAs were identified by *in silico* analysis to be flanked by a BHB motif with a folding energy lower than −9. Introns of tRNAs tRNA^*Trp*^ and tRNA^*Met*^ could be identified, as could circular 16S and 23S rRNA precursors. Columns: left site/right site: position of the BHB splice site; intron length: length of the respective intron; folding energy: folding energy of the BHB motif (in kcal/mol); gene: gene in which the intron is located.*

### The Splicing Endonuclease Processes a Pre-16S and the Newly Identified mRNA Substrate *in vitro*

**FIGURE 9:**
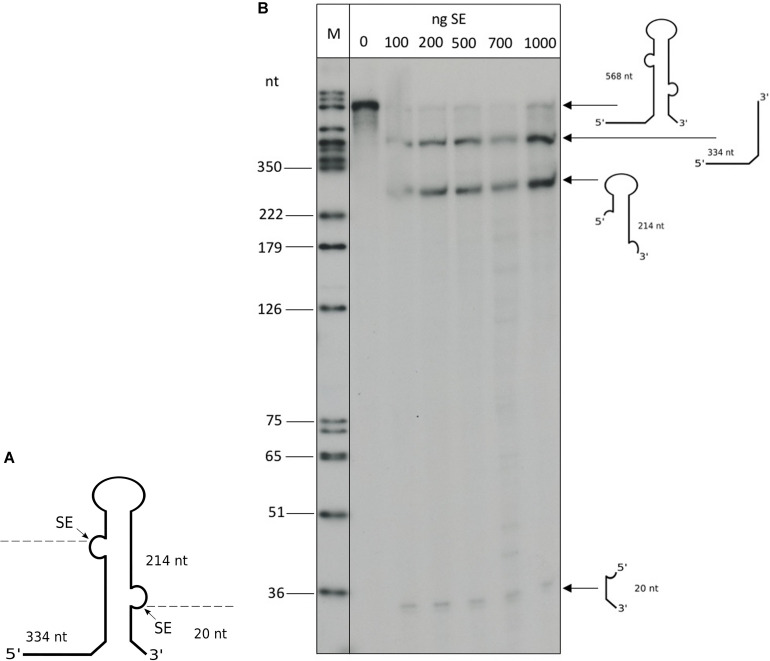
*In vitro* processing of a truncated 16S precursor. **(A)** Schematic drawing of the truncated 16S rRNA precursor used for the *in vitro* reaction: 1,450 nucleotides of the internal part of the 16S rRNA were deleted. Arrows and dashed lines indicate the SE splice sites and processing products. The resulting fragment sizes are given in nucleotides. **(B)** *In vitro* processing was performed with recombinant SE and the radioactively labeled substrate. Different amounts of SE protein were used (between 100 and 1,000 ng), as indicated at the top of the lanes. A DNA size marker is given on the left. The 568 nt long precursor is processed by the SE, resulting in three fragments: a 334 nt leader fragment, a 214 nt 16S rRNA loop fragment and a 20 nt trailer fragment. SE processing products can be observed beginning at 100 ng. Processing fragments are shown schematically on the right.

**FIGURE 10:**
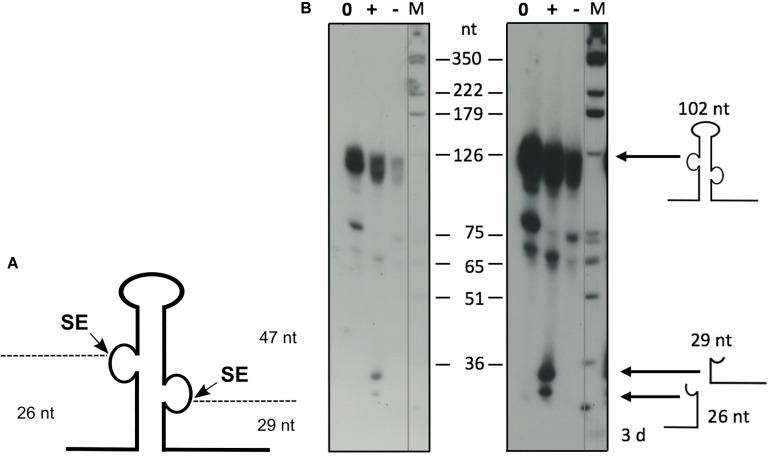
*In vitro* processing of the newly identified BHB containing RNA. **(A)** Schematic drawing of the precursor substrate. As a substrate for the *in vitro* processing reaction, the 5′ UTR of HVO_1309 was used, encompassing the intron flanked by the BHB motif with additional nucleotides up- and downstream. **(B)** Processing reaction. The substrate was transcribed *in vitro* and labeled throughout with [α−−^32^P]-UTP. *In vitro* processing was performed with recombinant SE, and RNAs were separated by 8% PAGE. Samples were incubated for 60 min. A DNA size marker is given at the sides, and sizes are shown in the middle (M). A control reaction without enzyme was performed at the beginning and end of the reaction time (indicated as NC0 and NC60 for 0 and 60 min of incubation, respectively). Samples with enzyme were incubated for 60 min (SE). Left panel: short exposure, right panel: long exposure. The precursor RNA has a length of 102 nucleotides, and the processing fragments are 26, 29, and 47 nucleotides long. Substrate and processing products are shown schematically at the side.

The *Haloferax* splicing endonuclease can be expressed in *E. coli* and processes intron-containing tRNAs effectively *in vitro* (Thompson and Daniels, 1990). To confirm that the ribosomal RNA precursors are substrates for SE, we processed a truncated 16S rRNA precursor *in vitro* with recombinant SE (Figure 9). To confirm the activity of the recombinant SE, a control reaction with the intron containing the tRNA^Trp^ precursor was performed that showed efficient processing (data not shown). The full length 16S rRNA precursor is more than 1,600 nucleotides long and such long precursors are suboptimal substrates for *in vitro* processing assays. Therefore, we generated a 568 nt long rRNA substrate, that had most of the mature rRNA part deleted, leaving only the 5′ and 3′ ends of the 16S rRNA and the BHB motif (Figure 9A). Incubation with the recombinant SE showed that this substrate is indeed efficiently processed (Figure 9B). To test whether the newly identified BHB motif found in the 5′ UTR of HVO_1309 (Table 3) is cleaved by SE, we incubated the respective precursor in an *in vitro* assay with recombinant SE (Figure 10), which revealed that this substrate was processed. The two products flanking the BHB are clearly visible (26 nt and 29 nt). The central fragment (47 nt) is either running slower and visible at approximately 65 nucleotides (a fragment of this size is also visible in the control reaction) or degraded.

## Discussion

### The Splicing Endonuclease Processes tRNAs, rRNAs, and a mRNA

Compared to bacteria and eukarya, only a few ribonucleases have been characterized in archaea. Furthermore, the ribonucleases which catalyze many key processing steps in cellular reactions remain unknown. It has been suggested that the few currently known archaeal ribonucleases may have a broader substrate spectrum and catalyze more processing steps than their bacterial and eukaryal homologs (Hölzle et al., 2012). For instance the *H. volcanii* tRNase Z – an endonuclease known for catalyzing tRNA 3′ processing (Schiffer et al., 2002; Späth et al., 2008)- has been shown to have an additional function besides tRNA processing; it is also involved in maturation of 5S ribosomal RNA (Hölzle et al., 2008).

Here, we aimed to identify all biological substrates of the tRNA splicing endonuclease in *H. volcanii* by applying the CRISPRi approach to repress *endA* expression to determine whether SE processes additional substrates beyond the known tRNA intron substrates. The growth of *endA* CRISPRi cells was severely affected, confirming the important cellular role of SE. We confirmed that all three intron containing tRNAs were down-regulated in the *endA* CRISPRi strain and that precursor tRNAs accumulated in the CRISPRi strain, establishing that the splicing endonuclease catalyses intron removal *in vivo*. In addition, we showed that the BHB motifs flanking the two larger ribosomal RNAs are also cleaved by SE *in vivo*. The processing of the 16S ribosomal RNA precursor by the SE could be confirmed with an *in vitro* processing experiment. This is in line with the observed processing of ribosomal RNAs in *Sulfolobus solfataricus* and *Methanolobus psychrophilus*, where maturation is also performed by the SE (Ciammaruconi and Londei, 2001; Qi et al., 2020).

To identify additional SE substrates, we employed circRNA-seq that identifies among others, the circular introns that are products of the splicing reaction. The rRNA and two of the tRNA circular introns were detected, and only the 31 nucleotide intron from tRNA^Gln^ was not found. It has been reported that circRNA-seq has a bias against shorter circRNAs (Danan et al., 2012) which might explain this observation. CircRNA-seq identified an additional circular RNA derived from the 5′ UTR of HVO_1309, and splicing removes 46 nucleotides from the 192 nucleotide long 5′ UTR. 5′ UTRs are known from bacteria/eukarya to be used as regulatory elements. 5′ UTRs can bind small regulatory RNAs or proteins to regulate the expression of downstream genes. They can also fold into specific structures binding distinct ligands, thereby also regulating downstream genes. Removal of the 46 nucleotides could eliminate a protein or sRNA binding site or inhibit/allow a specific structure to form. In archaea, only a few examples of interactions of proteins with 5′ UTRs have been reported (Daume et al., 2017; Li et al., 2019). The entire transcribed sequence of the HVO_1309 gene is conserved in haloarchaea, suggesting that the sequence of the 5′ UTR might be important. Since HVO_1309 is down-regulated in the CRISPRi strain, removal of the intron seems to be important for efficient expression of HVO_1309. Processing of the BHB sites in the 5′ UTR of HVO_1309 was confirmed by the *in vitro* assay. To date, only one example of an additional BHB intron has been reported in archaea situated in the *cbf5* mRNA in crenarchaeota (Watanabe et al., 2002; Yokobori et al., 2009). Whether the intron has a specific function is not yet known.

### Repression of the *endA* Gene Expression Results in Regulation of a Plethora of Genes

Comparison of the *endA* CRISPRi transcriptome with the wild type transcriptome showed that a plethora of genes are regulated upon repression of *endA*. Many tRNAs are affected by repression, suggesting that there is feedback from the defect in processing intron-containing tRNAs and perhaps even pre-rRNAs. The down-regulated tRNAs decode preferentially used codons; thus, depletion of these tRNAs surely has an impact on translation in general. However, little is known about how tRNA and rRNA expression are co-regulated and whether tRNA levels are co-regulated in archaea. In addition, it is difficult to assess the direct effects of inefficient tRNA and rRNA intron splicing and secondary effects that are due to low concentrations of mature tRNAs and rRNAs.

### *endA*/*trpS1* Operon Structure

The *endA* gene is located upstream of the *trpS1* gene, and the genes overlap with four nucleotides. We showed that *trpS1* is transcribed together with *endA* but it is also separately transcribed from independent promoters into a monocistronic mRNA. This is confirmed by the transcriptome data that show that the *trpS1* mRNA concentrations are not down-regulated in the CRISPRi strain. The expression of *trpS1* can be regulated from two promoters located in the *endA* ORF which allows regulation of *trpS1* expression independent of *endA* expression. This setup is another example of the independent regulation of genes that are part of the same operon (Berkemer et al., 2020).

*trpS1* codes for tryptophanyl-tRNA synthetase and is therefore important for loading tRNA^Trp^ with the aminoacyl group. Reduced levels of mature tRNA^Trp^ and therefore less tRNA to be loaded with an aminoacyl group might lead to a slight up-regulation of *trpS1* expression as a feedback mechanism.

## Materials and Methods

The strains, plasmids and primers used are listed in Supplementary Table 3.

### Growth of Strains

*Haloferax volcanii* strain HV30 was grown under aerobic conditions at 45°C in Hv-YPC medium or HV-Min medium containing the respective supplements (Allers et al., 2004). *E. coli* strain DH5α (Invitrogen^TM^, Thermo Fisher Scientific) was grown aerobically at 37°C in 2YT medium (Miller, 1972).

### Growth Experiment

*Haloferax volcanii* cells were grown in glass test tubes under shaking at 45°C in triplicates. The OD_650 nm_ was measured at different time points.

### RT-PCR

TRIzol^TM^ Reagent (Invitrogen^TM^, Thermo Fisher Scientific) was used to isolate total RNA from *H. volcanii* cells in the early exponential growth phase (OD_650_: ∼0.3). RNA was treated with RQ1 RNAfree DNase (Promega) to remove residual DNA. To investigate whether *endA* and *trpS1* are transcribed as bicistronic or two monocistronic transcripts, cDNA synthesis was carried out with 2 μg of DNA-free total RNA, RevertAid Reverse Transcriptase and random hexamer primers (Thermo Fisher Scientific). The subsequent PCR was performed with the gene-specific primers endAtrpS1_fw and endAtrpS1_rev to amplify the overlap of *endA* and *trpS1*. The primers trpS1intfw/rev were used to compare the signal of the *trpS1* transcript to the signals from the overlapping section. PCR products were separated on a 0.8% agarose gel in TAE buffer.

### qRT-PCR

#### Determination of the *endA* mRNA Concentrations

To determine the relative amount of *endA* transcript, RNA was isolated from *H. volcanii* cells in exponential growth phase from strains carrying only a control plasmid (HV30 × pTA232) or a plasmid expressing a spacer against the transcription start site of the *endA* gene (HV30 × pTA232SEa1-a4). Ten micrograms of total RNA was treated with 30 μl of RQ1 DNase (Promega), and cDNA synthesis was performed using random hexamer primers and RevertAid Reverse Transcriptase (Thermo Fisher Scientific). The quantitative PCRs were performed with the KAPA^TM^ SYBR fast Mastermix for Roche LightCycler^®^ Kapa Biosystems and the LightCycler^®^ 480 System (Roche). The primer sets used were q_endA_fw2 and q_endA_rev2 to amplify the *endA* transcript, q_tsgA3_fw2 and q_tsgA3_rev2 for tsgA3 and q_trmB1_fw2 and q_trmB1_rev2 for trmB1. Cycling conditions were as follows: initial denaturation for 5 min at 95°C; 40 cycles of denaturation for 15 s at 95°C, annealing of primers for 10 s at 59°C and elongation for 10 s at 72°C; followed by a melting curve determination for 5 s at 95°C and 1 min at 65°C. The reaction was completed by cooling for 30 sec at 40°C. qPCRs were carried out in triplicate in three independent experiments. Normalization of *endA* levels was obtained by using *tsgA3* and *trmB1* as references. Relative levels were calculated with the Roche LightCycler^®^ software according to the E-Method.

#### Determination of the Relative Abundance of Pre-rRNAs

Total RNA from logarithmically growing cells, cDNA synthesis and qRT-PCR analysis were essentially performed as described previously (Knüppel et al., 2017). For cDNA synthesis primers oHv040 (16S rRNA), oHv042 (23S rRNA) and oHv390 (Tfb) were used. The following primer pair combinations were used for qRT-PCR: oHv040/oHv039 (Hv_circ-pre-16S rRNA), oHv041/oHv042 (Hv_circ-pre-23S rRNA) oHv040/oHv200 (Hv_5’ extended-pre-16S rRNA) oHv042/oHv201 (Hv_5’ extended-pre-23S rRNA), oHv040/oHv205 (Hv_total 16S rRNA), oHv042/oHv206 (Hv_total 23S rRNA), and oHv391/oHv392 (Tfb) (Jüttner et al., 2020). Relative quantification analysis was performed using a comparative analysis software module (Rotor-gene 6 – Corbett Research/Qiagen). Relative levels were calculated according to the 2^–ΔΔCT^ method (Livak and Schmittgen, 2001) using the transcription initiation factor TFB (HVO_0226) mRNA level as reference. Relative RNA levels were normalized to WT. To ensure accuracy of the data, experiments were performed using biological triplicates (three CRISPRi transformants) and technical triplicates of serial dilutions (minimum two dilutions) of the cDNA samples were run.

#### CRISPRi

crRNAs against the region around the transcription start side of the *endA* gene were synthesized by Invitrogen GeneArt Gene Synthesis (Thermo Fisher Scientific). The plasmids carry a synthetic promoter and terminator for *H. volcanii* (Maier et al., 2015), tRNA-like-elements that flank the crRNA, and the crRNA gene that contains an 8 nt 5′ handle and the 36 nt spacer. The complete insert was cut from the plasmid with *Kpn*I and *Bam*HI and ligated with the *Haloferax* shuttle vector pTA232, yielding plasmids pTA232-SEa1 to 4. HV30 was transformed with one of the generated plasmids pTA232-SEa1 to 4 or the control plasmid pTA232. Cells were grown in Hv-Min selection medium containing tryptophan and uracil and harvested at selected OD_650 nm_ values. The repression effect was determined by northern blot analysis and qRT-PCR.

#### Analysis of Differentially Expressed Genes

RNA isolated from CRISPRi and control cultures was treated with TURBO^TM^ DNase (Invitrogen^TM^, Thermo Fisher Scientific) to remove all genomic DNA that was carried over during RNA preparation. Ten micrograms of total RNA was treated with 20 μl (40 units) of TURBO^TM^ DNase in a volume of 200 μl. Ten micrograms of DNA-free RNA was sequenced with next generation sequencing by Vertis Biotechnologie AG. The quality of raw sequencing reads was checked using FastQC version 0.11.4 (Andrews, 2010), and adaptor sequences and low-quality reads were trimmed using Cutadapt version 1.10 (Martin, 2011). Read mapping was performed using segemehl version 0.2.0 (Hoffmann et al., 2009; Otto et al., 2014). This was done for reads of three replicates from the wild type and three replicates from the CRISPRi mutant. The numbers of mapped and unmapped reads are listed in Table 4.

**TABLE 4:** Reads mapped to genome for the analysis of differentially expressed genes.

Sample	Replica	Total reads	Mapped reads	Uniquely mapped	Un-mapped	% of mapped reads
Wild type	S1	21,418,618	20,445,547	14,927,255	973,071	95.46%
	S2	21,816,231	20,725,427	14,408,066	1,090,804	95.00%
	S3	22,918,081	22,051,753	16,240,084	866,328	96.22%
CRISPRi	S1	22,626,113	21,810,284	17,903,446	815,829	96.39%
	S2	20,067,042	19,250,373	15,557,489	816,669	95.93%
	S3	21,902,393	21,043,653	16,769,061	858,740	96.08%

*The number of reads obtained (column total reads) and mapped (columns mapped reads, uniquely mapped reads, unmapped reads) from the different samples are shown.*

After the mapping, htseq-count (Anders et al., 2015) was used to calculate read counts as an input to DESeq2 (Love et al., 2014). DESeq2 was then applied to the data with the wild type sample as the control and the CRISPRi samples as the condition.

The resulting down- and upregulated genes are listed in Supplementary Tables 1, 2.

#### CircRNA-Seq and Detection of BHB Elements

RNA was isolated and treated as described above. To enrich circular RNAs the ribodepleted RNA samples were digested with Ribonuclease R (Lucigen, United States). RNA was then fragmented using ultrasound (4 pulses of 30 s each at 4°C). For cDNA synthesis oligonucleotide adapters were ligated to the 5′ and 3′ ends of the RNA fragments. First-strand cDNA synthesis was performed using M-MLV reverse transcriptase and the 3′ adapter as the primer. The resulting cDNAs were PCR-amplified using a high-fidelity DNA polymerase. The cDNA was purified using the Agencourt AMPure XP kit (Beckman Coulter Genomics) and analyzed by capillary electrophoresis. The cDNA pools were sequenced on an Illumina NextSeq 500 system using a 1 × 75 bp read length. The quality of raw sequencing reads was checked using FastQC version 0.11.4 (Andrews, 2010), and adaptor sequences and low-quality reads were trimmed using Cutadapt version 1.10 (Martin, 2011). Read mapping was performed using segemehl version 0.2.0 (Hoffmann et al., 2009; Otto et al., 2014) with the split-read option such that split reads and circularized sequences were additionally reported. This was done for reads of 3 replicates from the wild type and 3 replicates from the CRISPRi mutant. The numbers of mapped and unmapped reads are listed in the Table 5.

**TABLE 5:** Reads mapped to genome for CircRNA-seq.

Sample	Replica	Total reads	Mapped reads	Uniquely mapped	Un-mapped	% of mapped reads
Wild type	S1	13,356,856	13,086,209	11,602,090	270,647	97.97%
	S2	11,955,747	11,718,382	10,305,229	237,365	98.01%
	S3	14,669,440	14,457,586	11,515,934	211,854	98.56%
CRISPRi	S1	9,902,921	9,724,986	8,982,553	177,935	98.20%
	S2	11,089,425	10,868,626	10,090,045	220,799	98.01%
	S3	13,426,209	13,247,376	12,346,730	178,833	98.67%

*The number of reads obtained (column total reads) and mapped (columns mapped reads, uniquely mapped reads, unmapped reads) from the different samples are shown.*

Mapped reads were processed using samtools version 1.3 (Li et al., 2009). To calculate genome coverage and intersections of data sets, we used bedtools (bedtools v2.26.0) (Quinlan and Hall, 2010). Splice sites reported after the mapping were filtered such that only splice sites with a coverage of at least two that appear in all three replicates were kept. A pair of splice sites (left and right) describe the location of an intron. Various reported introns differ by only a few bases in their start or end coordinates. Such sites were merged if their distance was at most 10 nt. The coordinates of the site with the highest coverage were kept for further analyses. The numbers and varieties of linear and circularized introns in the wild type and CRISPRi mutant do not differ significantly; however, their coverage shows large differences regarding circularized introns (Table 6). The coverage is higher for putative introns in CRISPRi, which can be explained by lower splicing activity such that coverage refers to unspliced fragments, whereas the wild type introns undergo splicing and thus degradation more quickly.

**TABLE 6:** Introns in wild type and CRISPRi strains.

	Wild type			CRISPRi		
		
	Linear	Circula-rized	Total	Linear	Circula-rized	Total
Number	85	12	97	80	9	89
Average coverage	956.722	2,437.394	1,139.898	1,995.882	4,351.267	2,234.067

*Numbers and and average coverage of linear and circularized introns in wild type and CRISPRi strains is shown. The coverage is higher for putative introns in CRISPRi displaying the lower splicing activity.*

The search for BHB motifs was conducted using covariance models of known BHB motifs in *Haloferax* and programs of the infernal suite version 1.1 (Nawrocki and Eddy, 2013) for the detection of new motifs. BHB motifs are secondary structures that are formed out of two remotely located parts around the intron and act as a signal for the splicing machinery. The covariance model consists of a multiple sequence alignment of known BHB motifs and a consensus secondary structure. As the intronic sequences are not part of the motif and differ for each of the elements, sequences around the splice sites are cut and glued together such that intronic sequences are modeled as inserts. The program cmbuild from the infernal suite was used to create the covariance model. To set the focus on the secondary structure, the options –eset 0 and –hand were used. The model is shown below, where square brackets show base pairs and “b” denotes the bulge positions. Splice sites occur inside the bulges. Thus, areas around the splice sites are extracted from the sequences, where “∼” stands for the intronic sequence, which is not part of the model and thus is modeled as an insertion.


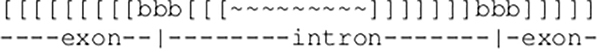


Reported splice sites are then used to extract sequences around the splice sites themselves to restrict the search space to a reasonable number of sequences and positions. As the BHB motif is a relatively small motif without sequence conservation, restriction of the search space is necessary for the current type of covariance models which only report hits with an overall good score. However, unmatched positions receive a bad score; thus, unrestricted searches would result in mostly badly scored hits. The program cmalign was used to apply the BHB covariance model to the set of target sequences. Here, a global alignment of the sequences to the model was used, to force the program to map sequences to the BHB motif instead of creating insertions or deletions. The results of cmalign were then additionally checked to only keep sequences where the splice site was located inside the bulge region. Another step was to use RNAcofold from ViennaRNA package [version 2.0, (Lorenz et al., 2011)] to check if sequences predicted to form a BHB motif truly fold into the predicted structure. RNAcofold additionally outputs the folding energy of the secondary structure motif, which indicates stability and thus probable occurrences of the motif.

#### Northern Blot Analysis

Total RNA was isolated from *H. volcanii* cells in the early exponential growth phase (OD_650 nm_: ∼0,3) with TRIzol^TM^ Reagent (Invitrogen^TM^, Thermo Fisher Scientific). DNase treatment to remove residual DNA was performed with RQ1 RNase-Free DNase (Promega). Ten micrograms of total RNA was separated by 8% PAGE and transferred to a nylon membrane (Hybond-N+, GE Healthcare). The membrane was hybridized with radioactively labeled oligonucleotide probes. Probes were labeled at the 5′ end by T4-polynucleotide kinase (PNK; Thermo Fisher Scientific) and [γ-^32^P]-ATP. To quantify the RNA, imaging plates (BAS-MS, Fujifilm) were exposed to radioactivity on the hybridized membranes and analyzed with the FLA-3000 scanner (GE Healthcare; software BASreader 3.14). Signal intensity was measured with ImageJ and set in relation to the signal of 5S rRNA that was detected and used as a loading control. The proportion of RNA is given as a percentage from cells expressing a targeting spacer and those carrying a control plasmid. The amount of RNA in the control strains was set to 100%, and the amounts in strains expressing a targeting spacer were calculated in relation to this value. Northern blot analyses were performed with biological triplicates of the control and CRISPRi strains.

#### *In vitro* Processing

The splicing endonuclease was expressed from the plasmid pTA28a(+)-*endA* in *E. coli* BL21Ai cells as fusion protein with an N-terminal His-tag. Expression of the protein was induced with 2 g/l arabinose and 1 mM IPTG at OD_600 nm_ 0.6 for 3 h at room temperature and under constant shaking. Cells were lysed by sonification (Sonofier 250, Branson Ultrasonics). After centrifugation, a His-tag purification was carried using the supernatant and N-terminal His-tag and Poly-Prep^®^ Chromatography Columns (Bio-Rad) with Protino^®^ Ni-NTA Agarose (Macherey-Nagel). The purity of the recombinant protein was confirmed by Coomassie stained SDS-PAGE gels and western blot. Templates for *in vitro* processing were cloned containing putative introns and exons of different lengths. The PCR product consisted of the T7 promoter and the respective genomic region. Primers for amplification were as follows: T7ptRNATrp and 3-tTrpTrailer, 1309-fw and 1309-rev and 5-T7prom-16SPrim, 3-*Xba*I- Leader-Trailer 16SPrim, 5-*Xba*I-16S3Trailer and 3-*Kpn*I-16S3Trailer. PCR products were generated by amplification with *Pfu* DNA Polymerase (Promega), treated with *Pfu* DNA Polymerase for 30 min at 72°C to generate blunt ends and separated on a 1% agarose gel. These DNA templates were used for *in vitro* transcription using T7 RNA Polymerase (New England Biolabs) in the presence of [α-^32^P]-UTP. After transcription, substrates were separated on a 8% PAA-TBE-urea-gel. RNAs were recovered from the gel by overnight elution at 4°C in elution buffer containing 0.5 M ammonium acetate, 0.1 mM EDTA and 0.1% SDS at pH 5. For *in vitro* processing, RNA substrates were denatured in SE buffer (10× SE buffer: 400 mM Tris pH 7.5, 25 mM spermidine) at 80°C for 5 min and renatured for 30 min at 21°C. Reactions with and without enzyme were carried out at 37°C and samples were taken at different time points. RNA was extracted from the processing reactions by phenol-/chloroform and precipitated with ethanol. RNA was separated on an 8%-PAA-TBE-urea-gel and transferred to filter paper and a photosensitive film was exposed to the gel.

## Data Availability Statement

The data were deposited in ENA with study accessions numbers: PRJEB40302 (primary) and ERP123923 (secondary).

## Author Contributions

TS, SBk, SBn, and MW did the experiments and data curation. AM conceptualized the project. TS, SBk, SF-C, PS, and AM wrote the original draft. SF-C, PS, and AM reviewed the draft, edited the draft, and provided the resources and funding. All authors contributed to the article and approved the submitted version.

## Conflict of Interest

The authors declare that the research was conducted in the absence of any commercial or financial relationships that could be construed as a potential conflict of interest.

## Footnotes

1 The TACK superphylum encompasses Thaumarchaeota, Aigarchaeota, Crenarchaeota Bathyarchaeota and Korarchaeota.

2 ARMAN: Archaeal Richmond Mine Acidophilic Nanoorganism.

3 hBHBh′: a canonical BHB splicing motif with the two outer helices having at least two Watson-Crick base pairs; h, the helix closing the 3-nt bulge on the exonic side; h′, the helix closing the 3-nt bulge on the intronic side.

4 http://www.kazusa.or.jp/codon/

## References

[B1] Allers T. Ngo H. P. Mevarech M. Lloyd R. G. (2004). Development of additional selectable markers for the halophilic archaeon *Haloferax volcanii* based on the leuB and trpA genes. *Appl. Environ. Microbiol.* 70 943–953. 10.1128/aem.70.2.943-953.2004 14766575 PMC348920

[B2] Anders S. Pyl P. T. Huber W. (2015). HTSeq–a Python framework to work with high-throughput sequencing data. *Bioinformatics* 31 166–169. 10.1093/bioinformatics/btu638 25260700 PMC4287950

[B3] Andrews S. (2010). *FastQC: A Quality Control Tool for High Throughput Sequence Data*.

[B4] Armbruster D. W. Daniels C. J. (1997). Splicing of intron-containing tRNATrp by the archaeon *Haloferax volcanii* occurs independent of mature tRNA structure. *J. Biol. Chem.* 272 19758–19762. 10.1074/jbc.272.32.19758 9242634

[B5] Babski J. Haas K. A. Nather-Schindler D. Pfeiffer F. Forstner K. U. Hammelmann M. (2016). Genome-wide identification of transcriptional start sites in the haloarchaeon *Haloferax volcanii* based on differential RNA-Seq (dRNA-Seq). *BMC Genomics* 17:629. 10.1186/s12864-016-2920-y 27519343 PMC4983044

[B6] Berkemer S. J. Maier L. K. Amman F. Bernhart S. H. Wortz J. Markle P. (2020). Identification of RNA 3 ends and termination sites in *Haloferax volcanii*. *RNA Biol.* 17 663–676. 10.1080/15476286.2020.1723328 32041469 PMC7237163

[B7] Ciammaruconi A. Londei P. (2001). In vitro processing of the 16S rRNA of the thermophilic archaeon *Sulfolobus solfataricus*. *J. Bacteriol.* 183, 3866–3874. 10.1128/JB.183.13.3866-3874.2001 11395449 PMC95268

[B8] Clouet d’Orval B. Bortolin M. L. Gaspin C. Bachellerie J. P. (2001). Box C/D RNA guides for the ribose methylation of archaeal tRNAs. The tRNATrp intron guides the formation of two ribose-methylated nucleosides in the mature tRNATrp. *Nucleic Acids Res.* 29 4518–4529. 10.1093/nar/29.22.4518 11713301 PMC92551

[B9] Clouet-d’Orval B. Batista M. Bouvier M. Quentin Y. Fichant G. Marchfelder A. (2018). Insights into RNA-processing pathways and associated RNA-degrading enzymes in Archaea. *FEMS Microbiol. Rev.* 42 579–613. 10.1093/femsre/fuy016 29684129

[B10] Danan M. Schwartz S. Edelheit S. Sorek R. (2012). Transcriptome-wide discovery of circular RNAs in Archaea. *Nucleic Acids Res.* 40 3131–3142. 10.1093/nar/gkr1009 22140119 PMC3326292

[B11] Daume M. Uhl M. Backofen R. Randau L. (2017). RIP-Seq Suggests Translational Regulation by L7Ae in &lt;em&gt;Archaea&lt;/em&gt. *mBio* 8:e00730-17. 10.1128/mBio.00730-17 28765217 PMC5539422

[B12] Fujishima K. Sugahara J. Miller C. S. Baker B. J. Di Giulio M. Takesue K. (2011). A novel three-unit tRNA splicing endonuclease found in ultrasmall Archaea possesses broad substrate specificity. *Nucleic Acids Res.* 39 9695–9704. 10.1093/nar/gkr692 21880595 PMC3239211

[B13] Haas K. A. (2016). *Untersuchung des CRISPR-Cas-Systems und der RNase G/E in Archaeen.* Ph.D. thesis, Ulm University, Ulm.

[B14] Hölzle A. Fischer S. Heyer R. Schütz S. Zacharias M. Walther P. (2008). Maturation of the 5S rRNA 5’ end is catalyzed in vitro by the endonuclease tRNase Z in the archaeon *H. volcanii*. *RNA* 14 928–937. 10.1261/rna.933208 18369184 PMC2327364

[B15] Hoffmann S. Otto C. Kurtz S. Sharma C. M. Khaitovich P. Vogel J. (2009). Fast mapping of short sequences with mismatches, insertions and deletions using index structures. *PLoS Comput Biol.* 5:e1000502. 10.1371/journal.pcbi.1000502 19750212 PMC2730575

[B16] Hölzle A. Stoll B. Schnattinger T. Schöning U. Tjaden B. Marchfelder A. (2012). tRNA-like elements in *Haloferax volcanii*. *Biochimie* 94 940–946. 10.1016/j.biochi.2011.12.002 22178322

[B17] Jevtić Ž Stoll B. Pfeiffer F. Sharma K. Urlaub H. Marchfelder A. (2019). The response of *Haloferax volcanii* to salt and temperature stress: a proteome study by label-free mass spectrometry. *Proteomics* 19:e1800491. 10.1002/pmic.201800491 31502396

[B18] Joardar A. Gurha P. Skariah G. Gupta R. (2008). Box C/D RNA-guided 2’-O methylations and the intron of tRNATrp are not essential for the viability of *Haloferax volcanii*. *J. Bacteriol.* 190 7308–7313. 10.1128/jb.00820-08 18757532 PMC2580716

[B19] Jüttner M. Weiß M. Ostheimer N. Reglin C. Kern M. Knüppel R. (2020). A versatile cis-acting element reporter system to study the function, maturation and stability of ribosomal RNA mutants in archaea. *Nucleic Acids Res.* 48 2073–2090. 10.1093/nar/gkz1156 31828323 PMC7038931

[B20] Kjems J. Garrett R. A. (1991). Ribosomal RNA introns in archaea and evidence for RNA conformational changes associated with splicing. *Proc. Natl. Acad. Sci. U.S.A.* 88 439–443. 10.1073/pnas.88.2.439 1899138 PMC50826

[B21] Kleman-Leyer K. Armbruster D. W. Daniels C. J. (1997). Properties of *H. volcanii* tRNA intron endonuclease reveal a relationship between the archaeal and eucaryal tRNA intron processing systems. *Cell* 89 839–847. 10.1016/s0092-8674(00)80269-x 9200602

[B22] Knüppel R. Kuttenberger C. Ferreira-Cerca S. (2017). Toward time-resolved analysis of RNA metabolism in archaea using 4-thiouracil. *Front. Microbiol.* 8:286. 10.3389/fmicb.2017.00286 28286499 PMC5323407

[B23] Li H. Handsaker B. Wysoker A. Fennell T. Ruan J. Homer N. (2009). The sequence alignment/map format and SAMtools. *Bioinformatics* 25, 2078–2079. 10.1093/bioinformatics/btp352 19505943 PMC2723002

[B24] Li H. Trotta C. R. Abelson J. (1998). Crystal structure and evolution of a transfer RNA splicing enzyme. *Science* 280 279–284. 10.1126/science.280.5361.279 9535656

[B25] Li J. Zhang B. Zhou L. Qi L. Yue L. Zhang W. (2019). The archaeal RNA chaperone TRAM0076 shapes the transcriptome and optimizes the growth of *Methanococcus maripaludis*. *PLoS Genet.* 15:e1008328. 10.1371/journal.pgen.1008328 31404065 PMC6705878

[B26] Livak K. J. Schmittgen T. D. (2001). Analysis of relative gene expression data using real-time quantitative PCR and the 2(-Delta Delta C(T)) method. *Methods* 25 402–408. 10.1006/meth.2001.1262 11846609

[B27] Lorenz R. Bernhart S. H. Zu Siederdissen C. H. Tafer H. Flamm C. Stadler P. F. (2011). ViennaRNA Package 2.0. *Algorithms Mol. Biol.* 6:26. 10.1186/1748-7188-6-26 PMC3319429 22115189

[B28] Love M. I. Huber W. Anders S. (2014). Moderated estimation of fold change and dispersion for RNA-seq data with DESeq2. *Genome Biol.* 15:550. 10.1186/s13059-014-0550-8 25516281 PMC4302049

[B29] Lykke-Andersen J. Garrett R. A. (1997). RNA-protein interactions of an archaeal homotetrameric splicing endoribonuclease with an exceptional evolutionary history. *EMBO J.* 16 6290–6300. 10.1093/emboj/16.20.6290 9321408 PMC1326313

[B30] Maier L. K. Stachler A.-E. Saunders S. J. Backofen J. Marchfelder A. (2015). An active immune defense with a minimal CRISPR (clustered regularly interspaced short palindromic repeats) RNA and without the Cas6 protein. *J. Biol. Chem.* 290, 4192–4201. 10.1074/jbc.M114.617506 25512373 PMC4326828

[B31] Martin M. (2011). Cutadapt removes adapter sequences from high-throughput sequencing reads. *EMBnet. J.* 17, 10–12. 10.14806/ej.17.1.200

[B32] Marck C. Grosjean H. (2003). Identification of BHB splicing motifs in intron-containing tRNAs from 18 archaea: evolutionary implications. *RNA* 9 1516–1531. 10.1261/rna.5132503 14624007 PMC1370505

[B33] Miller J. H. (1972). *Experiments in Molecular Genetics*. New York: Cold Spring Harbour Laboratory Press.

[B34] Mitchell M. Xue S. Erdman R. Randau L. Söll D. Li H. (2009). Crystal structure and assembly of the functional Nanoarchaeum equitans tRNA splicing endonuclease. *Nucleic Acids Res.* 37 5793–5802. 10.1093/nar/gkp537 19578064 PMC2761253

[B35] Mullakhanbhai M. F. Larsen H. (1975). *Halobacterium volcanii* spec. nov., a Dead Sea halobacterium with a moderate salt requirement. *Arch. Microbiol.* 104 207–214. 10.1007/bf00447326 1190944

[B36] Nakamura M. Sugiura M. (2007). Translation efficiencies of synonymous codons are not always correlated with codon usage in tobacco chloroplasts. *Plant J.* 49 128–134. 10.1111/j.1365-313X.2006.02945.x 17144890

[B37] Nawrocki E. P. Eddy S. R. (2013). Infernal 1.1: 100-fold faster RNA homology searches. *Bioinformatics* 29 2933–2935. 10.1093/bioinformatics/btt509 24008419 PMC3810854

[B38] Oren A. (2008). Microbial life at high salt concentrations: phylogenetic and metabolic diversity. *Saline Syst.* 4:2. 10.1186/1746-1448-4-2 18412960 PMC2329653

[B39] Otto C. Stadler P. F. Hoffmann S. (2014). Lacking alignments? The next-generation sequencing mapper segemehl revisited. *Bioinformatics* 30, 1837–1843. 10.1093/bioinformatics/btu146 24626854

[B40] Pfeiffer F. Broicher A. Gillich T. Klee K. Mejia J. Rampp M. (2008). Genome information management and integrated data analysis with HaloLex. *Arch. Microbiol.* 190 281–299. 10.1007/s00203-008-0389-z 18592220 PMC2516542

[B41] Qi L. Li J. Jia J. Yue L. Dong X. (2020). Comprehensive analysis of the pre-ribosomal RNA maturation pathway in a methanoarchaeon exposes the conserved circularization and linearization mode in archaea. *RNA Biol.* 17 1427–1441. 10.1080/15476286.2020.1771946 32449429 PMC7549664

[B42] Quinlan A. R. Hall I. M. (2010). BEDTools: a flexible suite of utilities for comparing genomic features. *Bioinformatics* 26, 841–842. 10.1093/bioinformatics/btq033 20110278 PMC2832824

[B43] Schiffer S. Rösch S. Marchfelder A. (2002). Assigning a function to a conserved group of proteins: the tRNA 3’-processing enzymes. *EMBO J.* 21 2769–2777. 10.1093/emboj/21.11.2769 12032089 PMC126033

[B44] Schulze S. Adams Z. Cerletti M. De Castro R. Ferreira-Cerca S. Fufezan C. (2020). The Archaeal Proteome Project advances knowledge about archaeal cell biology through comprehensive proteomics. *Nat. Commun.* 11:3145 10.1038/s41467-020-16784-7 PMC7305310 32561711

[B45] Singh S. K. Gurha P. Tran E. J. Maxwell E. S. Gupta R. (2004). Sequential 2’-O-methylation of archaeal pre-tRNATrp nucleotides is guided by the intron-encoded but trans-acting box C/D ribonucleoprotein of pre-tRNA. *J. Biol. Chem.* 279 47661–47671. 10.1074/jbc.m408868200 15347671

[B46] Späth B. Schubert S. Lieberoth A. Settele F. Schütz S. Fischer S. (2008). Two archaeal tRNase Z enzymes: similar but different. *Arch. Microbiol.* 190 301–308. 10.1007/s00203-008-0368-4 18437358

[B47] Stachler A. E. Marchfelder A. (2016). Gene repression in Haloarchaea using the CRISPR (clustered regularly interspaced short palindromic repeats) - Cas I-B system. *J. Biol. Chem.* 291 15226–15242. 10.1074/jbc.m116.724062 27226589 PMC4946936

[B48] Stachler A. E. Schwarz T. S. Schreiber S. Marchfelder A. (2019). CRISPRi as an efficient tool for gene repression in archaea. *Methods* 172 76–85. 10.1016/j.ymeth.2019.05.023 31150759

[B49] Steiger M. A. Jackman J. E. Phizicky E. M. (2005). Analysis of 2’-phosphotransferase (Tpt1p) from Saccharomyces cerevisiae: evidence for a conserved two-step reaction mechanism. *RNA* 11 99–106. 10.1261/rna.7194605 15611300 PMC1370695

[B50] Taboada B. Ciria R. Martinez-Guerrero C. E. Merino E. (2012). ProOpDB: prokaryotic operon database. *Nucleic Acids Res.* 40 D627–D631. 10.1093/nar/gkr1020 22096236 PMC3245079

[B51] Tang T. H. Rozhdestvensky T. S. d’Orval B. C. Bortolin M. L. Huber H. Charpentier B. (2002). RNomics in Archaea reveals a further link between splicing of archaeal introns and rRNA processing. *Nucleic Acids Res.* 30 921–930. 10.1093/nar/30.4.921 11842103 PMC100335

[B52] Thompson L. D. Daniels C. J. (1990). Recognition of exon-intron boundaries by the *Halobacterium volcanii* tRNA intron endonuclease. *J. Biol. Chem.* 265 18104–18111. 1698785

[B53] Watanabe Y. Yokobori S. Inaba T. Yamagishi A. Oshima T. Kawarabayasi Y. (2002). Introns in protein-coding genes in Archaea. *FEBS Lett.* 510 27–30. 10.1016/s0014-5793(01)03219-7 11755525

[B54] Yokobori S. Itoh T. Yoshinari S. Nomura N. Sako Y. Yamagishi A. (2009). Gain and loss of an intron in a protein-coding gene in Archaea: the case of an archaeal RNA pseudouridine synthase gene. *BMC Evol. Biol.* 9:198. 10.1186/1471-2148-9-198 19671140 PMC2738675

