# Supplementary material for: Splicing Endonuclease Is an Important Player in rRNA and tRNA Maturation in Archaea
Source: Front Microbiol. 2020 Nov 20;11:594838. doi: 10.3389/fmicb.2020.594838 (PMC7714728; doi:10.3389/fmicb.2020.594838)
Supplement: Supplementary file 1 [file Data_Sheet_1.PDF]

Supplementary Data

Supplementary Figures page 2

Supplementary Tables page 4

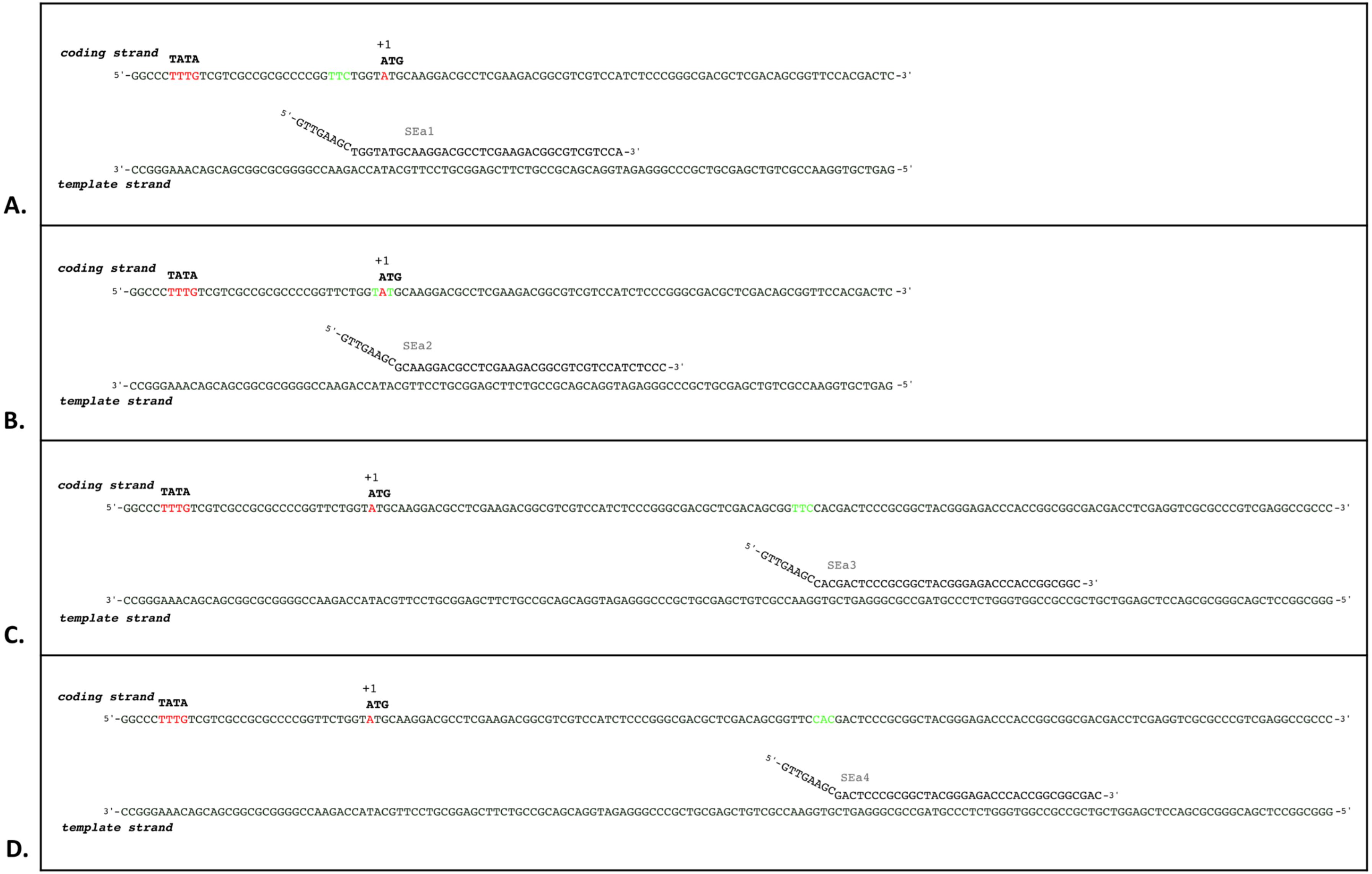

Supplementary Figure 1. Detailed view of the binding location of all four crRNAs. Four different crRNAs were designed to bind to the *endA* template strand. Binding sites are located at the transcription start site and in the 5’ region of the ORF. The promoter is indicated with red letters and "TATA". The transcription and translation start site are identical, indicated with +1 and the red letter A of the ATG, resulting in a leaderless *endA* mRNA. All four crRNAs are 44 nucleotides long, consisting of 8 nucleotides long 5´ leader sequence (repeat sequence) and 36 nucleotides spacer sequence, the latter defines the binding site. **A.** The first nucleotide of crRNA SEa1 binds at position -4 relative to the TSS. **B.** The binding site of crRNA SEa2 starts at position +3 relative to the TSS. **C.** The first nucleotide of crRNA SEa3 binds at position +61 relative to the TSS. **D.** The binding site of crRNA SEa4 starts at position +64 relative to the TSS.

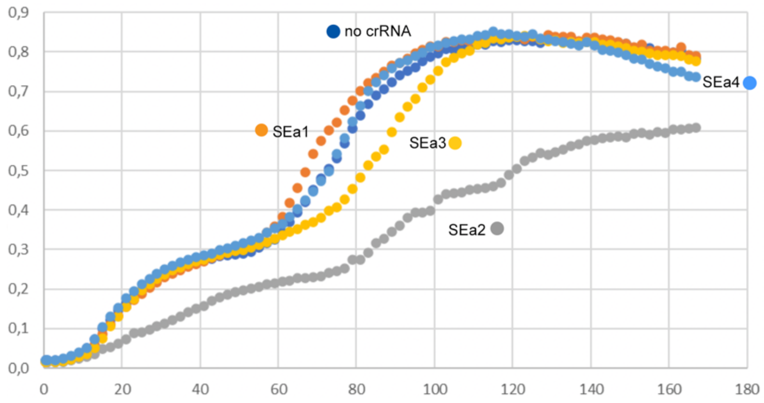

Supplementary Figure 2. *endA* CRISPRi repression with four different crRNAs. Expression of crRNA SEa2 results in a severe lag phase (grey dots, SEa2). Expression of crRNA SEa3 results in slower growth in late exponential phase (yellow dots, SEa3). Expression of crRNAs SEa1 and SEa4 does not have a negative effect on cell growth (orange dots, SEa1; light blue dots, SEa4). As control cells were transformed with a plasmid tha did not express a crRNA (dark blue dots, no crRNA).

Supplementary Tables

Supplementary Table 1. Genes down-regulated in *endA*-CRISPRi strain. Genes that show a log_2 fold down-regulation of at least 2 are listed. Fourty-one genes are down-regulated by the CRISPRi repression of the *endA* gene. The *endA* gene itself is down-regulated by 7.94 fold. Ten of the 41 affected genes are tRNA genes. All three intron encoding tRNA genes are downregulated. Eleven genes encoding proteins of unknown functions are downregulated and six RNAs of unknown functions. Column "gene": HVO gene number; column log_2: log_2 fold change; column “p-value”: statistical confidence measure; column “p_{adj}”: adjusted p-value; column "annotation": gene annotation.

gene	log_2	p-value	p_{adj}	annotation
HVO_2952	-7.9	4E-60	2E-56	tRNA splicing endonuclease
HVO_0575	-4.7	2E-27	2E-24	homolog to NAD-dependent epimerase/ dehydratase
HVO_2519_T	-4.6	2E-38	2E-35	$tRNA^{Met}$, also termed HVO_3026
HVO_2566	-3.8	9E-09	4E-07	$tRNA^{Gly}$, also termed HVO_3052
HVO_0864_T	-3.5	2E-20	1E-17	$tRNA^{Gln}$, also termed HVO_t3070
HVO_B0149	-3.3	4E-16	1E-13	oleate hydratase
HVO_1800	-2.9	5E-16	1E-13	hypothetical protein
HVO_1561	-2.9	1E-09	7E-08	conserved hypothetical protein
HVO_2804_T	-2.9	1E-05	2E-04	$tRNA^{Thr}$ (TGT)
HVO_1276_T	-2.7	2E-42	3E-39	$tRNA^{Trp}$, also termed HVO_t3075
HVO_0640	-2.6	1E-04	2E-03	$tRNA^{Ser}$, also termed HVO_3028
HVO_0036	-2.5	2E-21	9E-19	conserved hypothetical protein
HVO_0907s	-2.4	2E-01	NA	RNA with unknown function
HVO_A0526	-2.4	4E-02	1E-01	hypothetical protein
HVO_1725s	-2.4	8E-12	8E-10	RNA with unknown function
HVO_1358	-2.4	2E-13	2E-11	receiver box response regulator
HVO_A0127	-2.4	6E-15	1E-12	tryptophan-tRNA ligase
HVO_0012	-2.4	1E-09	6E-08	$tRNA^{Gln}$, also termed HVO_3066
HVO_B0276	-2.3	1E-01	3E-01	DMT superfamily transport protein
HVO_A0632s	-2.3	8E-06	2E-04	RNA with unknown function
HVO_1052	-2.3	2E-18	6E-16	transcription initiation factor TFB
HVO_A0319	-2.2	3E-09	1E-07	hypothetical protein
HVO_1883	-2.2	3E-10	2E-08	DUF457 family protein
HVO_A0257s	-2.2	1E-16	3E-14	RNA with unknown function
HVO_1732	-2.1	3E-06	7E-05	$tRNA^{Cys}$, also termed HVO_3042
HVO_1355s	-2.1	7E-05	9E-04	RNA with unknown function
HVO_1355	-2.1	4E-23	2E-20	UPF0058 family protein
HVO_1533	-2.1	7E-05	9E-04	small CPxCG-related zinc finger protein
HVO_1309	-2.1	3E-14	4E-12	peptidase M24 family protein (homolog to Xaa-Pro dipeptidase)
HVO_0037	-2.1	5E-20	2E-17	conserved hypothetical protein
HVO_B0336	-2.1	2E-01	NA	probable hydrolyase (homolog to altronate dehydratase) (nonfunctional)
HVO_2492	-2	1E-12	1E-10	conserved hypothetical protein
HVO_2354s	-2	7E-17	2E-14	RNA with unknown function
HVO_2381	-2	2E-05	3E-04	UPF0272 family protein
HVO_A0634	-2	1E-06	3E-05	peptidase M20 family protein (homolog to succinyl-diaminopimelate desuccinylase)
HVO_2599	-2	1E-09	7E-08	metallophosphoesterase domain protein
HVO_A0168	-2	8E-05	1E-03	HTH-10 family transcription regulator
HVO_2567	-2	3E-04	3E-03	$tRNA^{Gly}$, also termed HVO_3053
HVO_2352	-2	2E-05	3E-04	$tRNA^{Ile}$, also termed HVO_3048

Supplementary Table 2.

Genes up-regulated in the *endA* CRISPRi strain. Genes that show a log_2 fold up-regulation of at least 2 are listed. 102 genes are up-regulated upon CRISPRi repression of the *endA* gene. 15 genes encode proteins that are involved in information storage and processing, 54 genes code for proteins involved in metabolism. Four RNAs with unknown function are up-regulated as well as 22 genes encoding proteins of unknown function. Column "gene": HVO gene number; column log_2: log_2 fold change; column “p-value”: statistical confidence measure; column “p_{adj}”: adjusted p-value; column "annotation": gene annotation.

gene	log_2	p-value	p_{adj}	annotation
HVO_1696	7	1E-07	4E-06	L-lactate permease
HVO_B0044	4.8	1E-03	1E-02	siderophore biosynthesis protein IucA
HVO_B0042	4.7	1E-03	9E-03	probable 1,3-diaminopropane N-3-monooxygenase, IucD
HVO_B0045	4.5	6E-03	3E-02	diaminobutyrate decarboxylase
HVO_1228	4.2	1E-03	1E-02	DUF5059 domain / halocyanin domain protein
HVO_B0043	4.1	2E-03	1E-02	probable N4-hydroxy-1-aminopropane O-acetyltransferase
HVO_B0047	4	2E-03	1E-02	ABC-type transport system periplasmic substrate-binding protein
HVO_B0046	3.9	4E-03	3E-02	diaminobutyrate--2-oxoglutarate aminotransferase
HVO_B0197	3.8	2E-03	1E-02	ABC-type transport system permease protein
HVO_1697	3.6	3E-06	8E-05	FAD-dependent oxidoreductase (GlcD/DLD_GlcF/GlpC domain fusion protein)
HVO_B0198	3.6	3E-03	2E-02	ABC-type transport system periplasmic substrate-binding protein
HVO_B0041	3.5	3E-06	8E-05	siderophore biosynthesis protein IucC
HVO_0538	3.4	1E-04	2E-03	SirR/DtxR family transcription regulator
HVO_B0056	3.4	2E-06	5E-05	probable ferredoxin (4Fe-4S)
HVO_0751	3.4	7E-02	NA	conserved hypothetical protein
HVO_1463	3.2	2E-05	3E-04	oxidoreductase (homolog to thioredoxin-disulfide reductase)
HVO_B0144	3.1	6E-06	1E-04	ABC-type transport system periplasmic substrate-binding protein
HVO_B0186	3.1	1E-05	2E-04	ABC-type transport system permease protein (probable substrate dipeptide/oligopeptide)
HVO_B0150	3	3E-05	4E-04	ABC-type transport system periplasmic substrate-binding protein
HVO_1991	3	4E-05	6E-04	ABC-type transport system periplasmic substrate-binding protein
HVO_2038	3	1E-05	2E-04	ABC-type transport system periplasmic substrate-binding protein
HVO_B0065	3	4E-07	1E-05	thioredoxin domain protein
HVO_B0063	3	3E-05	5E-04	CbtB family protein
HVO_B0053	3	7E-05	9E-04	DUF3209 family protein
HVO_B0057	3	8E-06	2E-04	cobalt-factor-III C17-methyltransferase
HVO_2066	2.9	8E-07	2E-05	conserved hypothetical protein
HVO_0540	2.9	9E-06	2E-04	conserved hypothetical protein
HVO_2142	2.9	4E-04	4E-03	small CPxCG-related zinc finger protein
HVO_B0055	2.9	2E-05	3E-04	conserved hypothetical protein
HVO_1539	2.8	3E-04	3E-03	glycerol-3-phosphate dehydrogenase subunit B
HVO_A0558	2.8	1E-04	1E-03	ABC-type transport system periplasmic substrate-binding protein
HVO_0539	2.8	2E-04	2E-03	conserved hypothetical protein
HVO_A0155	2.7	5E-04	4E-03	DUF4212 family protein
HVO_0778s	2.6	3E-11	3E-09	RNA with unknown function
HVO_A0152	2.6	2E-05	3E-04	conserved hypothetical protein
HVO_1705	2.6	1E-04	1E-03	ABC-type transport system periplasmic substrate-binding protein (probable substrate iron)
HVO_B0054	2.6	3E-05	5E-04	sirohydrochlorin cobaltochelatase
HVO_B0058	2.6	3E-07	9E-06	cobalt-factor-III C17-methyltransferase
HVO_B0059	2.6	4E-06	1E-04	cobalt-precorrin-5A hydrolase

HVO_B0060	2.6	4E-05	6E-04	cobalt-precorrin-4 C11-methyltransferase
HVO_B0052	2.6	2E-04	2E-03	PQQ repeat protein
HVO_1542	2.6	7E-09	3E-07	conserved hypothetical protein
HVO_2737	2.6	7E-16	2E-13	50S ribosomal protein L8e
HVO_B0048	2.5	4E-05	6E-04	cobalt-precorrin-7 C5-methyltransferase
HVO_B0185	2.5	3E-05	5E-04	ABC-type transport system permease protein (probable substrate dipeptide/oligopeptide)
HVO_0852_A	2.5	NA	NA	conserved hypothetical protein
HVO_2034	2.5	6E-05	8E-04	ABC-type transport system permease protein (probable substrate sugar)
HVO_2976	2.5	9E-06	2E-04	carbon starvation protein CstA
HVO_B0050	2.5	9E-06	2E-04	ATP-dependent cobaltochelatase subunit CobN
HVO_B0064	2.5	3E-05	4E-04	CbtA family protein
HVO_1796	2.5	2E-03	1E-02	conserved hypothetical protein
HVO_A0551	2.4	2E-05	4E-04	acyl-CoA synthetase
HVO_2558	2.4	1E-10	1E-08	30S ribosomal protein S3
HVO_0778	2.4	1E-05	2E-04	thermosome subunit 3
HVO_1760	2.4	4E-03	2E-02	ABC-type transport system ATP-binding protein
HVO_B0062	2.4	9E-05	1E-03	cobalt-precorrin-6B C15-methyltransferase (decarboxylating)
HVO_2998	2.3	7E-04	6E-03	homoserine O-acetyltransferase
HVO_B0066	2.3	2E-05	3E-04	Lrp/AsnC family transcription regulator
HVO_0534	2.3	2E-05	3E-04	ABC-type transport system ATP-binding protein (probable substrate sugar)
HVO_2742	2.3	2E-04	2E-03	5-methyltetrahydropteroyltriglutamate--homocystei ne S-methyltransferase (methionine synthase II)
HVO_1612	2.3	8E-10	5E-08	DUF3179 family protein
HVO_1083s	2.3	7E-02	2E-01	RNA with unknown function
HVO_1488	2.3	1E-06	4E-05	D-gluconate dehydratase
HVO_0894	2.3	6E-04	5E-03	acyl-CoA synthetase
HVO_1362	2.3	2E-01	NA	conserved hypothetical protein
HVO_1541	2.3	1E-05	2E-04	glycerol kinase
HVO_2554	2.2	3E-11	3E-09	50S ribosomal protein L14
HVO_0133	2.2	1E-05	2E-04	thermosome subunit 1
HVO_B0061	2.2	2E-04	2E-03	cobalt-factor-II C20-methyltransferase
HVO_1386	2.2	1E-02	6E-02	conserved hypothetical protein
HVO_1706	2.2	1E-04	1E-03	ABC-type transport system permease protein (probable substrate iron)
HVO_0532	2.2	1E-06	3E-05	ABC-type transport system permease protein (probable substrate sugar)
HVO_A0489	2.2	2E-03	1E-02	homolog to homocitrate synthase
HVO_B0184	2.2	4E-04	4E-03	ABC-type transport system periplasmic substrate-binding protein (probable substrate dipeptide/oligopeptide)
HVO_2740	2.2	8E-15	1E-12	nucleoside-diphosphate kinase
HVO_1764	2.2	4E-03	2E-02	conserved hypothetical protein
HVO_2707	2.2	6E-04	5E-03	GFO family oxidoreductase
HVO_0537	2.1	8E-02	2E-01	conserved hypothetical protein
HVO_A0541	2.1	2E-03	1E-02	ABC-type transport system periplasmic substrate-binding protein
HVO_A0518	2.1	3E-01	NA	homolog to methylmalonyl-CoA epimerase (nonfunctional)
HVO_1759	2.1	8E-03	4E-02	ABC-type transport system permease protein
HVO_2110	2.1	3E-03	2E-02	IclR family transcription regulator
HVO_B0049	2.1	5E-04	5E-03	cobalt-precorrin-8 methylmutase
HVO_1538	2.1	5E-04	5E-03	glycerol-3-phosphate dehydrogenase subunit A
HVO_2556	2.1	3E-10	2E-08	ribonuclease P protein component 1
HVO_A0340	2.1	5E-01	NA	hypothetical protein
HVO_2548	2.1	2E-13	2E-11	50S ribosomal protein L6

HVO_0530	2.1	4E-03	2E-02	ABC-type transport system periplasmic substrate-binding protein (probable substrate sugar)
HVO_2560	2.1	2E-18	6E-16	30S ribosomal protein S19
HVO_2555	2.1	3E-11	3E-09	30S ribosomal protein S17
HVO_2073s	2.1	3E-11	3E-09	htsf_289
HVO_A0490	2	1E-03	8E-03	family 3 CoA transferase
HVO_A0492	2	6E-03	3E-02	ABC-type transport system permease protein (probable substrate sugar)
HVO_C0052	2	2E-05	3E-04	ISH4-type transposase ISHvo5 (nonfunctional)
HVO_0541	2	7E-03	4E-02	aconitate hydratase
HVO_2551	2	3E-13	3E-11	50S ribosomal protein L5
HVO_2738	2	1E-24	8E-22	30S ribosomal protein S28e
HVO_2562	2	1E-12	2E-10	50S ribosomal protein L23
HVO_1704	2	1E-03	1E-02	homolog to arabinopyranose mutase
HVO_2736s	2	3E-06	7E-05	RNA with unknown function
HVO_2405	2	1E-07	5E-06	ABC-type transport system ATP-binding protein (probable substrate nickel)
HVO_1262	2	NA	NA	probable NhaC-type sodium/proton antiporter

Supplementary Table 3. Strains, Plasmids and Oligonucleotides

Strains used in this study

Haloferax		
HV30	DS70(ΔpHV2), Δ*pyrE2*, Δ*leuB*, Δ*trpA*, Δ*cas3*, Δ*cas6*, Δ*bgaH*	(Stachler et al., 2019)
E. coli		
E. coli DH5a	F^-, Φ80dlacZΔM15, Δ(lacZYA-argF)U169, deoR, recA1, endA1, hsdR17(r_k^-, m_k^+), *phoA*, supE44, λ^-, thi-1, gyrA96, relA1	Invitrogen
E. coli BL21Ai	F^-, ompT, gal, dcm, $hsdS_B$(r_B^-, m_B^-), araB::T7RNAP- tetA, $TetA^R$	Invitrogen

Plasmids used in this study

pTA232	ColE1 ori, f1 ori, *lacZ*, Amp^R, *leuB*, pHV2 ori	(Allers et al., 2004)
pMA-RQ-SEa1	ColE1 ori, f1 ori, AmpR, *p.syn* promoter, crRNA SEa1, t.syn terminator	GeneArt
pMA-RQ-SEa2	ColE1 ori, f1 ori, AmpR, *p.syn* promoter, crRNA SEa2, t.syn terminator	GeneArt
pMA-RQ-SEa3	ColE1 ori, f1 ori, AmpR, *p.syn* promoter, crRNA SEa3, t.syn terminator	GeneArt
pMA-RQ-SEa4	ColE1 ori, f1 ori, AmpR, *p.syn* promoter, crRNA SEa4, t.syn terminator	GeneArt
pTA232-SEa1	ColE1 ori, f1 ori, lacZ, Amp^R, *leuB*, pHV2 ori, P.syn promoter, SEa1 construct, T.syn terminator	this study
pTA232-SEa2	ColE1 ori, f1 ori, lacZ, Amp^R, *leuB*, pHV2 ori, P.syn promoter, SEa2 construct, T.syn terminator	this study
pTA232-SEa3	ColE1 ori, f1 ori, lacZ, Amp^R, *leuB*, pHV2 ori, P.syn promoter, SEa3 construct, T.syn terminator	this study
pTA232-SEa4	ColE1 ori, f1 ori, lacZ, Amp^R, leuB, pHV2 ori, P.syn promoter, SEa4 construct, T.syn terminator	this study
pET28a	f1 ori, pBR322 ori, Kan^R, lacI, T7-Promotor, T7- Terminator, (6xHis-Tag (N/C-term.))	Novagen
pET28a(+)-*endA*	f1 ori, pBR322 ori, Kan^R, lacI, T7-Promotor, T7- Terminator, endA-gene 6xHis-Tag N-term.	this study
pUC18-16S-Leader-Trailer	pMB1 ori, lacZ, AmpR, leader and trailer of the 16S rRNA (HVO_2038)	(Haas, 2016)

Oligonucleotides

name	sequence	use
T7prom	TAATACGACTCACTATAGGG	

T7term	GCTAGTTATTGCTCAGCGG	colony screening
US	GTAACGCCAGGGTTTTCCC	
RS	CACAGGAAACAGCTATGAC	
tRNATrp Splice Junction	CCCCGATCGACTGATCTGGAGTCAGTCGCC	probe for $tRNA^{Trp}$ detection
5-endA-BamHI_2	cgtagatggatccATGCAAGGACGCCTCGAA	amplification of *endA* from gDNA for cloning of pET28a(+)-*endA*
3-endA-HindIII	gctacaagcttcTCATGGTGTGAGCCGACTGACC	
pT7Trpfw	TAATACGACTCACTATAGGGGGCTGTGGCCAAGC	cloning of $tRNA^{Trp}$ ivT template, without leader and trailer
3-$tRNA^{Trp}$	TGGGGCCGGAGGGATTTG	
T7ptRNA^{Trp}	TTAATACGACTCACTATAGGCAGACAGAACGAATC	cloning of pre-$tRNA^{Trp}$ ivT template; with leader and trailer
3-tTrpTrailer	AAAACCCTGCGAAAGGATT	
5-T7prom-16SPrim	GGAGATCTAATACGACTCACTATAGCGAAGAAGCGCA CGGGG GCAC	cloning of 16S-Leader-Trailer ivT template
3-XbaI-Leader-Trailer 16SPrim	TATATCTAGACAACCGGAATGGGTCGCGACC	
5-XbaI-16S3Trailer	TATATCTAGATGGATCACCTCCTAACG	
3-KpnI-16S3Trailer	TATAGGTACCGTGTTAGCCCTAGTAG	
tMetCAT_SplJunc	CCACGATCTCCGCATTATGAGTGCGGCGCT	probe for $tRNA^{Met}$ detection
tGlnTTG_SplJunc	CATAGCGGCCTTTGGAGCCGCTGACGGCGG	probe for $tRNA^{Gln}$ detection
trpS1intfwd	CGCTCATCGCGCTCGGGTTCGACCCCG	Test-PCR DNA-free RNA; comparison bicistronic / monocistronic transcript *endA* / *trpS1*
trpS1intrev	GCTTCTCGACCGCCGATTCGCGGACCG	
endAprobe3_fw	GATTTCCTCGTCTACCCCCG	Test-PCR DNA-free RNA
endAprobe3_rev	CGGTGTCGAAGTACGTCAGG	

HvoIB7int_fw	GTGATGCGCTCAACAGTTCG	Test-PCR DNA-free RNA
HvoIB7int_rev	CGCGAGGTCGTCTGATTCTT	
endAtrpS1 fw	TCGGCTCGGACTTCCGCGTCTAC	comparison bicistronic / monocistronic transcript *endA* / *trpS1*
endAtrpS1 rev	CGGCATGAAGCCCGAGAGGACG	
q_endA_fw2	GTCCGCAAGCGAATGGTTTT	primers for qPCR endA
q_endA_rev2	GTGAGCCGACTGACCGAAA	
q_tsgA3_fw2	GTGAAGAACCGACCCTCCTG	
q_tsgA3_rev2	CCGAGAGGTTCTGCAGTTGA	
q_trmB1_fw2	GGAGCACATCGTCTCTTCGT	
q_trmB1_rev2	TTTTGACCCGACATGCACCT	
2695_cDNA	GATTGCGCGCGATGAACGG	primers for RT-PCR SE substrates
2695_RT_FW	ACCGAAGAAACGACCGAA	
2695_RT_REV	GTCGGTTCTTCACGGC	
1374_cDNA	CGTCCGAAAGCGCCCCCGCGA	
1374_RT_FW	ATGGTAGGTGCTACCAAC	
1374_RT_REV	GTACTCCGCGTACGTGTA	
1542_cDNA	GATACCGTAGGCGAGGAAG	
1542_RT_FW	ACGACGGCGTTCATCGCG	
1542_RT_REV	GAGGATACCGGCTCCGAG	
2855_cDNA	CGGCGACCCGACGCGTCG	
2855_RT_FW	GCCGGAAGCGCAAGGG	
2855_RT_REV	ACGGCTCGCCCGCTC	
1882_cDNA	GTCGGAGCGGACGTGCGAG	
1882_RT_FW	TCCACGTCGACGCCGACC	
1882_RT_REV	GGTTCCGCCACGGCCGG	
1276T_cDNA	TGGGGCCGGAGGGATTTG	
1276T_RT_FW	GGGGCTGTGGCCAAGCC	
1276T_RT_REV	AACCCCCGATCGACTGA	
oHv039 - ci16S-Fw	CGAATCTGGGCTTCGCAAGG	primers for qPCR rRNA
oHv040 - ci16S-Rv	GTATGAACTCGTGCAACTAGC	
oHv041 - ci23S-Fw	CGATAGACTCGGGGTGTACGC	
oHv042 - ci23S-Rv	CAGCTTGGCACGTCCGTCATC	
oHv200 - Hv-revS1-001	CCTGCGGTACGCCGCAAGAC	
oHv201 - Hv-revS3-006	GCAGTACTCCACTCCGAAACG	
oHv205 - 16S rRNA_5’ end	ATTCCGGTTGATCCTGCCGG	
oHv206 - 23S rRNA_5' end	CTGTGCCAGCTGGTGGATAG	
oHv390 - TFB-RT-rv	CACGTACGTCTTGCGGAGCG	

oHv391 - TFB-qPCR-Fw	GAGTACCGCATCGACCACGG	
oHv392 - TFB-qPCR-Rv	TGATACGCGTCGAGCGGCCG	
oHv393 - SE-RT-Rv	TGGTGTGAGCCGACTGACCG	
oHv394 - SE-qPCR-Fw	CGGCGAGGTCGAACACCGGG	
oHv395 - SE-qPCR-Rv	AGTTCCGCGTCGAGGTCGGC	
T7ptRNATrp	TTAATACGACTCACTATAGGCAGACAGAACGAATC	Primers for generating ivT templates of potential SE substrates
3-tTrpTrailer	AAAACCCTGCGAAAGGATT	
SubA-fw	TTAATACGACTCACTATAGGCGGTCGCGGCGA	
SubA-rev	CTCTCGCGTCGCGGTCGGCG	
T7-2855-fw	ATAATACGACTCACTATAGGTTCATTATGTTATCGGAC	
2855-ivT-rev	GTCGTAAACTGCGCCGCCGCGCG	
1309-fw	TTAATACGACTCACTATAGGCGGTCGCGGCGA	
1309-rev	CTCTCGCGTCGCGGTCGGCG	
T7-a0041-fw	TAATACGACTCACTATAGGGTGCTCGAAAGCAG	
a0041_ivT-rev	GACGATTACGACCGACCCC	
T7-a0603-fw	TAATACGACTCACTATAGGGAGCCATCCGCACT	
a0603-ivT-rev	CGAGTGCTTGGTATCAAGT	
oHv039 - ci16S-Fw	CGAATCTGGGCTTCGCAAGG	
oHv040 - ci16S-Rv	GTATGAACTCGTGCAACTAGC	
oHv041 - ci23S-Fw	CGATAGACTCGGGGTGTACGC	
oHv042 - ci23S-Rv	CAGCTTGGCACGTCCGTCATC	
oHv200 - Hv-revS1-001	CCTGCGGTACGCCGCAAGAC	
oHv201 - Hv-revS3-006	GCAGTACTCCACTCCGAAACG	
oHv205 - 16S rRNA_5’ end	ATTCCGGTTGATCCTGCCGG	
oHv206 - 23S rRNA_5' end	CTGTGCCAGCTGGTGGATAG	
oHv390 - TFB-RT-rv	CACGTACGTCTTGCGGAGCG	
oHv391 - TFB-qPCR-Fw	GAGTACCGCATCGACCACGG	
oHv392 - TFB-qPCR-Rv	TGATACGCGTCGAGCGGCCG	
oHv393 - SE-RT-Rv	TGGTGTGAGCCGACTGACCG	
oHv394 - SE-qPCR-Fw	CGGCGAGGTCGAACACCGGG	
oHv395 - SE-qPCR-Rv	AGTTCCGCGTCGAGGTCGGC	

References

Allers, T., Ngo, H.P., Mevarech, M., and Lloyd, R.G. (2004). Development of additional selectable markers for the halophilic archaeon Haloferax volcanii based on the leuB and trpA genes. *Appl Environ Microbiol* 70(2)**,** 943-953.

Haas, K.A. (2016). *Untersuchung des CRISPR-Cas-Systems und der RNase G/E in Archaeen.* PhD, Ulm University.

Stachler, A.E., Schwarz, T.S., Schreiber, S., and Marchfelder, A. (2019). CRISPRi as an efficient tool for gene repression in archaea. *Methods*. doi: 10.1016/j.ymeth.2019.05.023.
